# Loss of mitochondrial transcription factor A in neural stem cells leads to immature brain development and triggers the activation of the integral stress response *in vivo*

**DOI:** 10.1371/journal.pone.0255355

**Published:** 2021-07-28

**Authors:** Rintaro Kuroda, Kaoru Tominaga, Katsumi Kasashima, Kenji Kuroiwa, Eiji Sakashita, Hiroko Hayakawa, Tom Kouki, Nobuhiko Ohno, Kensuke Kawai, Hitoshi Endo

**Affiliations:** 1 Department of Biochemistry, Jichi Medical University, Shimotsuke, Tochigi, Japan; 2 Department of Neurosurgery, Jichi Medical University, Shimotsuke, Tochigi, Japan; 3 Core Center of Research Apparatus, Jichi Medical University, Shimotsuke, Tochigi, Japan; 4 Department of Anatomy, Jichi Medical University, Shimotsuke, Tochigi, Japan; 5 Division of Ultrastructural Research, National Institute for Physiological Sciences, Okazaki, Aichi, Japan; Lewis Katz School of Medicine at Temple University, UNITED STATES

## Abstract

Mitochondrial dysfunction is significantly associated with neurological deficits and age-related neurological diseases. While mitochondria are dynamically regulated and properly maintained during neurogenesis, the manner in which mitochondrial activities are controlled and contribute to these processes is not fully understood. Mitochondrial transcription factor A (TFAM) contributes to mitochondrial function by maintaining mitochondrial DNA (mtDNA). To clarify how mitochondrial dysfunction affects neurogenesis, we induced mitochondrial dysfunction specifically in murine neural stem cells (NSCs) by inactivating Tfam. Tfam inactivation in NSCs resulted in mitochondrial dysfunction by reducing respiratory chain activities and causing a severe deficit in neural differentiation and maturation both in vivo and in vitro. Brain tissue from Tfam-deficient mice exhibited neuronal cell death primarily at layer V and microglia were activated prior to cell death. Cultured Tfam-deficient NSCs showed a reduction in reactive oxygen species produced by the mitochondria. Tfam inactivation during neurogenesis resulted in the accumulation of ATF4 and activation of target gene expression. Therefore, we propose that the integrated stress response (ISR) induced by mitochondrial dysfunction in neurogenesis is activated to protect the progression of neurodegenerative diseases.

## Introduction

Mitochondria are highly dynamic organelles that contribute to cellular energy metabolism by generating ATP through oxidative phosphorylation (OXPHOS) according to energy demands [1]. Recently, it was discovered that mitochondria play a significant role in cellular signaling and gene regulation through the production of intermediate metabolites and the proper amount of reactive oxygen species (ROS) [2–5]. Mitochondrial integrity is tightly maintained to execute various cellular activities and its dysregulation triggers mitochondrial dysfunction through the production of excess ROS and reduced ATP production. Mitochondrial dysfunction is associated with the pathogenesis of many diseases including age-related diseases [6–9]. Therefore, elucidating how mitochondrial integrity is maintained represents an important area of research.

During neurogenesis, neural stem cells (NSCs) are maintained by self-renewal and they differentiate into neuronal and glial lineages in a time-dependent manner [10–12]. Mitochondrial dynamics are modulated through fission and fusion cycles and are associated with the lineage stages of neurogenesis [13–16]. ATP production by mitochondria is important for maintaining neuronal function and activity, but it is severely compromised in mitochondrial disease patients [17–20]. Although it is known that mitochondria are maintained and their morphology is dynamically regulated throughout neurogenesis, the role of mitochondrial activity at each stage remains unknown.

Mitochondrial transcription factor A (TFAM), a nuclear-encoded factor, is essential for maintaining the copy number and structure of mitochondrial DNA (mtDNA) as well as the transcription and replication of mtDNA [20–24]. The expression of TFAM and the mtDNA copy number are proportional. Therefore, mitochondrial activity is strongly affected by TFAM levels in the mitochondria. A conditional TFAM knockout mouse model is useful for the study of mitochondria because mitochondrial activities can be downregulated by TFAM depletion. Loss of TFAM in murine forebrain neurons using neuron-specific CaMK II-Cre mice resulted in a late-onset mitochondrial neurodegeneration (MILON) phenotype [25]. Because massive neuronal cell death at end stages and excitotoxic stress conditions were observed in MILON mice, mitochondrial activities are important for maintaining proper neuronal activities in this model.

In this study, we examined the role of mitochondria during neurogenesis using mice exhibiting conditional Tfam inactivation in nestin-expressing NSCs. Tfam-deficient mice were viable and indistinguishable from control littermates at birth. However, after postnatal day 4, the Tfam-deficient mice exhibited severe growth retardation and died around day 10, possibly from a feeding deficiency. Neurons in Tfam-deficient mouse brain were immature and their mitochondria were swollen. NSCs cultured as neurospheres from Tfam-deficient mouse brains exhibited defective growth characteristics and their neural differentiation ability was severely compromised. ROS production from Tfam-deficient NSCs was reduced because of a reduction in mtDNA. Microglia was activated prior to apoptosis in Tfam-deficient brains. Finally, we showed that the integrated stress response (ISR) was activated in Tfam-deficient brains and NSCs in response to reduced mitochondrial activity. The results suggest that mitochondrial dysfunction caused by mtDNA defects induces ISR activation in the brain which prevents the progression of neurodegeneration.

## Materials and methods

### Mice

We used C57BL/6 mice in which the Tfam gene was specifically knocked out in neural stem cells (NSCs) [26]. Nestin is considered to be a neural stem cell marker which is expressed in NSCs after embryonic day 11.5 (E11.5) in mice [27]. Homozygous floxed Tfam females (Tfam^fl/fl^) were crossed with Tfam^fl/+^; Nestin-Cre^+^ males to produce mice in which the Tfam genes were knocked out in the NSCs and their lineage cells (Tfam^fl/fl^; Nestin-Cre^+^ indicated as Tfam cKO hereafter). All experiments were performed using Tfam cKO mice with their littermates as controls. The mice were maintained under controlled environmental conditions including a circadian rhythm consisting of a 12 h light and 12 h dark cycle with free access to standard mouse chow and water. All animal experiments were conducted in a humane manner with approval from the Institutional Animal Experiment Committee of Jichi Medical University and in accordance with the Institutional Regulation for Animal Experiments and the Fundamental Guidelines for Proper Conduct of Animal Experiment and Related Activities in Academic Research Institutions under the jurisdiction of the Ministry of Education, Culture, Sports, Science and Technology.

### Neurosphere culture

We sacrificed pregnant mice at gestational day 14.5 (E14.5) and removed the embryos [28, 29]. Forebrains (cerebellar cortex or ganglionic eminence) were dissected from the embryonic brains and triturated with a P1000 micropipette for dispersal into single cells. The dispersed cells (1 x10^6^ cells) were seeded into 60-mm low-attachment PrimeSurface (Sumilon) dishes and cultured in neural stem cell growing medium consisting of NeuroBasal medium, 2% B-27 without vitamin A, 2 mM glutamine, 20 ng/mL rhEGF (Pepro Tech), and 10 ng/mL rhFGF2 (Pepro Tech) in a humidified incubator containing 5% CO_2_. After 4–6 days, the cells aggregated, proliferated, and formed neurospheres. The neurospheres were collected and used for further experiments.

### Neurosphere formation assay

Neurosphere formation assay was performed as previously described [28]. Briefly, neurospheres formed from the primary cultures were dispersed into single cells using Accumax. The dispersed cells (1,000 cells/well) were seeded into Ultra-Low-Attachment Surface 96-well plates (Corning) in neural stem cell growth medium and cultured for 10 days. The diameter and number of neurospheres were determined using a KEYENCE BZ-X700 microscope and the neurospheres were categorized by size.

### BrdU incorporation assay

Neurospheres formed from primary culture were dispersed into single cells. The dispersed cells (2 × 10^5^ cells/well) were seeded into 12-well plates (Iwaki) using coverslips coated with ploy-L-ornithine and cultured overnight in neural stem cell growth medium. The cells were incubated with 10 μM BrdU in NSC growth medium for 2 h. The cells were then fixed with 70% ethanol for 20 min and treated with 2.5 N HCl for 20 min. After extensive washing with PBS, the coverslips were blocked in 5% fetal bovine serum (FBS) and 0.1% Triton X-100 in PBS and incubated with primary antibody against BrdU (1:200 BD Sciences 347580) overnight at 4°C. Following a 1-h incubation with secondary antibody (AlexaFluor488 1:1000 ThermoFisher), DAPI was used for nuclear staining. Coverslips were mounted with ProLong Gold antifade reagent (Invitrogen) and the images were captured using a KEYENCE BZ-X700 microscope. Image analysis was performed using Cell Analyzer software (Keyence).

### Differentiation assay

Neurospheres formed from primary culture were dispersed into single cells. The dispersed cells (2 × 10^5^ cells/well) were seeded into 12-well plates (Iwaki) using coverslips coated with ploy-L-ornithine in NeuroBasal differentiation medium, 2% B-27 with Vitamin A, 2 mM glutamine and 1% FBS and incubated for 7 days. The cells were fixed with 4% paraformaldehyde (PFA) in PBS for 10 min and blocked for 1 h with 5% FBS and 0.1% Triton X-100 in PBS. The cover slips were incubated with primary antibodies (Tuj1/beta-III Tubulin 1:2000 Abcam ab1820, GFAP 1:1000 Sigma G3893) overnight at 4°C followed by secondary antibodies (AlexaFluor488 1:1000 ThermoFisher, Cy3 1:1000 Amersham Pharmacia) 1 h. DAPI was used for nuclear staining. The coverslips were mounted with ProLong Gold antifade reagent (Invitrogen) and images were captured using a KEYENCE BZ-X700 microscope. Image analysis was performed with Cell Analyzer software (Keyence).

### Histology

Mice at postnatal day 0, 4, 8, or 9 (P0, P4, P8, or P9, respectively) were anesthetized by injection with pentobarbital into the peritoneal cavity and perfusion was performed with 4% PFA in PBS injected into the left cardiac ventricle. The brains were removed and stored in 4% PFA overnight and soaked in cryoprotection solution (20% sucrose in PBS) for 2 to 3 days. Brains were embedded in OCT compound and stored at −80°C until use. The coronal sections were prepared using a cryostat (Leica CM-3050S) set at a 14-mm thickness and mounted onto adhesive glass slides (Matsunami MAS-01). Hematoxylin and eosin (H&E) staining was performed according to standard procedures.

### Immunohistochemistry

For antigen retrieval, sections were soaked in 10 mM of citrate (pH6.0), boiled at 105°C for 5 min in an autoclave, and kept at room temperature for 30 min. After washing with PBS, the sections were permeabilized with 0.2% Triton X-100 in PBS and blocked with 5% FBS/0.1% Triton X-100 in PBS. The sections were incubated with antibodies in 5% FBS/0.1% Triton X-100 in PBS at 4°C overnight or at room temperature for 1 h. Secondary antibodies were incubated with the sections in 5% FBS/0.1% Triton X-100 in PBS at room temperature for 1 h. DAPI (1 μg/mL in PBS) was used for nuclear staining. The sections were mounted with ProLong Gold antifade reagent (Invitrogen) and images were captured using a KEYENCE BZ-X700. Image analysis was performed with Cell Analyzer software (Keyence). The antibodies used were: MAP2 (1:250, Proteintech 17490-1-AP), Cux1 (1:500 Santa Cruz sc-13024), Ctip2 (1:1000 Abcam 18465), MTCO1 (1:1000 Abcam ab14705), VDAC1/Porin (1:1000 Abcam ab15895), activated caspase 3 (1:2000 CST 9665), Iba1 (1:1000 Abcam ab178846), GFAP (1:2000 Sigma G3893).

### Golgi staining

The Super Golgi kit (Bioenno Tech, LLC) was used for staining sectioned brains following the instruction manual. Brains were coronally sectioned at 150-μm thickness using a vibratome.

### DNA and RNA extraction from mouse brains

Brains at P0, P4, P8 were isolated, quickly frozen in liquid nitrogen, and stored at −80°C until use. A portion of the frozen brains was used for DNA isolation using the DNeasy Blood & Tissue Kit (Qiagen) following the manufacturer’s instructions. Frozen brains or cells were homogenized in Trizol reagent by passing through a G21 needle and the isolated RNA was dissolved in nuclease free water. Total RNA (1 μg) was used to synthesize cDNA using the PrimeScript RT reagent kit (Takara).

### Quantitative PCR (qPCR)

Quantitative PCR (qPCR) was performed using Thunderbird SYBR (TOYOBO) and a Thermal Cycler Dice Real Time System II (TaKaRa). For relative copy number analysis of mtDNA, 10 ng of DNA extracted from the brains were assayed. Amplification levels of mtDNA were normalized to the nuclear gene encoding 18S ribosome RNA. We designed primer pair at cytochrome b coding region in mtDNA (MN964117.1, forward primer: 14882–14901 and reverse primer: 15010–15029). The sequences of the primer pairs were used, are shown in S1 Table.

qRT-PCR was performed using cDNAs synthesized from brain RNAs. A list of all primer sequences is provided in S2 Table. Relative expression was normalized to that of β-actin and calculations were done using the ΔΔCt method.

### Extracellular flux analyzer

Neurospheres formed from primary culture were dispersed into single cells. The cells (4 × 10^4^ cells/well) were seeded into Seahorse XFp plates coated with poly-L-ornithine in neural stem cell growth medium and incubated overnight. The medium was replaced with XF Base medium containing 10 mM glucose, 1 mM sodium pyruvate, and 2 mM glutamine and the assay plate was incubated for 1 h without a CO_2_ control. The Mito-stress test was performed on cells using the XFp flux analyzer (Agilent Tech.) and the oxygen consumption rate (OCR) and extracellular acidification rate (ECAR) were monitored following serial injection of oligomycin (1 μM), FCCP (1 μM), and rotenone/antimycin A mixture (0.5 μM).

### Measuring endogenous ROS levels

Single cells prepared from cultured neurospheres were seeded at 1 × 10^6^ cells/well on 6-well plates coated with poly-L-ornithine and incubated overnight. On the day of analysis by flow cytometry, ROS detector (5 μM of MitoSox Red) was incubated with the cells for 15 min at 37°C. The cells were removed from the plates using Accumax, washed once with 1 × Hanks solution, and suspended in 0.5 mL of growth medium. Prior to analysis by flow cytometry, the cells were passed through a 40-μm cell strainer to remove aggregated cells. The events were acquired on a BD LSRFortessa X-20 (BD Biosciences, Franklin Lakes, NJ) flow cytometer and analyzed using FlowJo software Ver. 9. 9. 6 (FlowJo LLC, Ashland, OR).

### Transmission electron microscopy

Mice at P10 were anesthetized using 0.01% pentobarbital, perfused with 0.1 M phosphate buffer (PB, pH 7.4), and fixed in 2% PFA/2.5% glutaraldehyde in 0.1 M PB. The brains were isolated, immersed in the fixative until subsequent processing, and sliced at a 500-μm thickness using a vibratome. Thereafter, the tissue pieces containing cerebral cortex were dissected out from the slices, and post-fixation was performed with 1% OsO_4_ solution in 0.1 M PB for 90 min. After dehydration, the samples were embedded in epoxy resin and sectioned at 70-nm thickness. The sections were stained by a double‐contrast method using 2% uranyl acetate and Reynolds lead citrate solution and analyzed using a Hitachi HT7700 transmission electron microscope (Hitachi High Technologies).

### Western blot analysis

Total cell lysates were prepared from cells or brains using RIPA buffer (20 mM Tris-HCl [pH 7.5], 150 mM NaCl, 1% sodium deoxycholate, 1% Triton X-100, and 0.1% SDS) supplemented with protease inhibitors (Calbiochem) and incubated with Benzonase nuclease (Novagen) at room temperature for 5 min. After sonication for 30 sec, the lysates were centrifuged at 20,000 × g for 10 min at 4°C to remove insoluble debris. Proteins were separated on 10% SDS-PAGE gels and transferred to nitrocellulose membranes. The membranes were blocked with 2% FBS/0.5% skim milk in PBST (0.05% tween 20) and probed with various antibodies at 4°C overnight. The bands were visualized using an ECL detection system (GE Healthcare) and analyzed with a LAS4000 mini Luminescent Image Analyzer (GE Healthcare). The band intensity was quantified by using ImageQuant TL (GE Healthcare). The antibodies used were: NDUFA9 (1:250 Abcam ab14713), SDHA (1:1000 Molecular Probe A-11142), UQCRC2 (1:500 Abcam ab14745), ATP5α (1:1000 Molecular Probe A-11144), Drp1 (1:500 Abcam ab56788), Mfn1 (1:500 Abcam ab104274), Mfn2 (1:500 Abcam ab124773), VDAC1/Porin (1:800 Abcam ab15895), ATF-4 (1:1000 CST 11815), β-actin (1:1000 MBL M177-3).

### Statistics

Statistical analyses were performed using a two-tailed t-test and differences were considered significant with p values of *p < 0.05, **p<0.01, or ***p<0.001.

## Results

### Tfam cKO mice are born at the expected ratio but exhibit postnatal lethality

**Fig 1:**
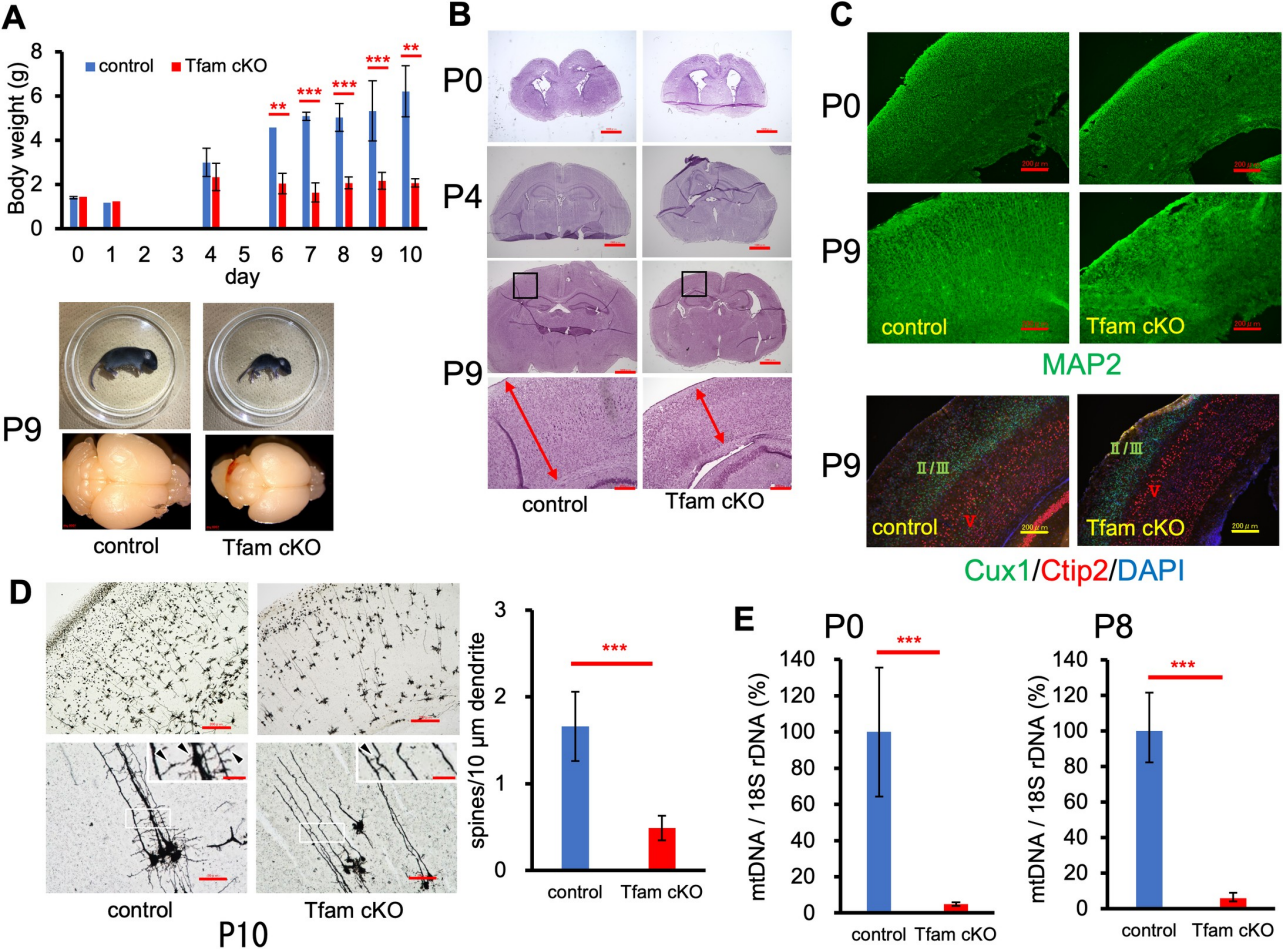
Tfam inactivation in neural stem cells (NSCs) results in postnatal growth retardation and neuronal maturation defects. (A) Measurement of body wight after birth. Body wight was measured on the indicated day. Representative images of mice at P9 are shown at the bottom. Graph shows means ± SD. At least n = 3 mice per group. **P < 0.01; ***P < 0.001. (B) Representative images stained with H&E at P0, P4, and P9. Enlarged view in the inset of P9 panels is shown at the bottom. Scale bars, 1000 μm, 200 μm in enlarged view. (C) Representative images of brains at P0 and P9 stained for MAP2 by immunofluorescence. Immunostaining of brains for layer-specific marker proteins, Cux1 (Layer II/III) and Ctip2 (layer V), at P9. DAPI was used for nuclear staining. Scale bars, 200 μm. (D) Representative images at low (upper images) and high (lower images) magnification of control (left) and Tfam cKO (right) brain sections with Golgi staining at P10. Areas marked with white rectangles are enlarged in insets showing dendritic arborization and spines (arrowheads). Scale bars, 200 μm for upper panels, 50 μm for lower panels, 20 μm for inset in lower panels. Quantification of the spine number per 10 μm dendrite. Graph shows means ± SD. ***P < 0.001. (E) Quantitative PCR analysis of relative mtDNA copy number in control and Tfam cKO brains at P0 and P8. Graph shows means ± SD. n = 3 mice per group. ***P < 0.001. At least two independent experiments were performed.

All mice were born with the expected Mendelian ratio independently of their genotypes and Tfam cKO (Tfam^fl/fl^; Nestin-Cre^+^) mice could not be distinguished from the others at birth. Because we were unable to distinguish any phenotypic differences between Tfam^fl/fl^; Nestin-Cre^-^ and Tfam^fl/+^; Nestin-Cre^-^ mice, we used these mice as controls. Growth retardation of Tfam cKO mice emerged with growth progression and became obvious at approximately postnatal day 4 (P4). Body weight of the mice did not increase after P4 and none of the Tfam cKO mice survived to P10. The body weight of the Tfam cKO mice was significantly less compared with that of their littermates after P6 and the brain sizes of the Tfam cKO mice were much smaller compared with that of their littermates at P9 (**Fig 1A**).

We conducted a morphological and histopathological examination for coronal sections of the postnatal brains. At P0, the thickness of the cortex at the anterior hippocampal region of Tfam cKO mice exhibited no difference compared with that of the control mice, whereas the cortex of Tfam cKO mice was much thinner compared with that of the controls at P9 (**Fig 1B**).

To evaluate neuron organization, we performed immunohistochemistry using an antibody against MAP2, a mature neuron marker. There was no obvious difference in MAP2 immunostaining in brains from Tfam cKO mice and controls at P0 (**Fig 1C**, upper panels). However, the alignment of neurites in the cortex of Tfam cKO brains was disorganized, whereas the alignment of neurites was well-organized in the cortex of the controls at P9 (**Fig 1C**, middle panels). In general, the mammalian neocortex is composed of six neuronal layers [30, 31]. We examined cortex layer formation in the Tfam cKO brains. We used antibodies against Cux1 and Ctip2 to detect neurons in layers II/III [32] and layer V [33], respectively [34]. Compared with layer V, the thickness of layer II/III was decreased in the Tfam cKO cortex compared with that of the control, although both Cux1- and Ctip2-positive neurons migrated correctly. This suggests that neuronal migration during neurogenesis was not disrupted in Tfam cKO mice (**Fig 1C**, bottom panels).

Because it was believed that the maturation of neurons and the organization of neuronal networks is affected in Tfam-deficient brains during neurogenesis, we examined dendritic arborization and dendritic spine formation in Tfam cKO brains at P10 by Golgi staining. As shown in **Fig 1D**, dendritic arborization were reduced and dendritic spines were diminished in Tfam cKO brains compared with control brains.

TFAM is an essential factor for mitochondrial genome maintenance. We next compared the relative amount of mtDNA in the Tfam cKO cortex with that of the control cortex. The relative amount of mtDNA in the Tfam cKO cortexes decreased to greater than ten-fold less compared with the controls at P0 and this pattern was the same at P8 (**Fig 1E**). It was surprising that the amount of mtDNA was significantly different between Tfam cKO and control mice because we did not observe any pathophysiological symptoms in Tfam cKO mice at P0.

### NSCs and/or neural progenitor cells (NPCs) from Tfam cKO mice have reduced potentials for proliferation and neural differentiation and gene expression of their mtDNAs decreases

**Fig 2:**
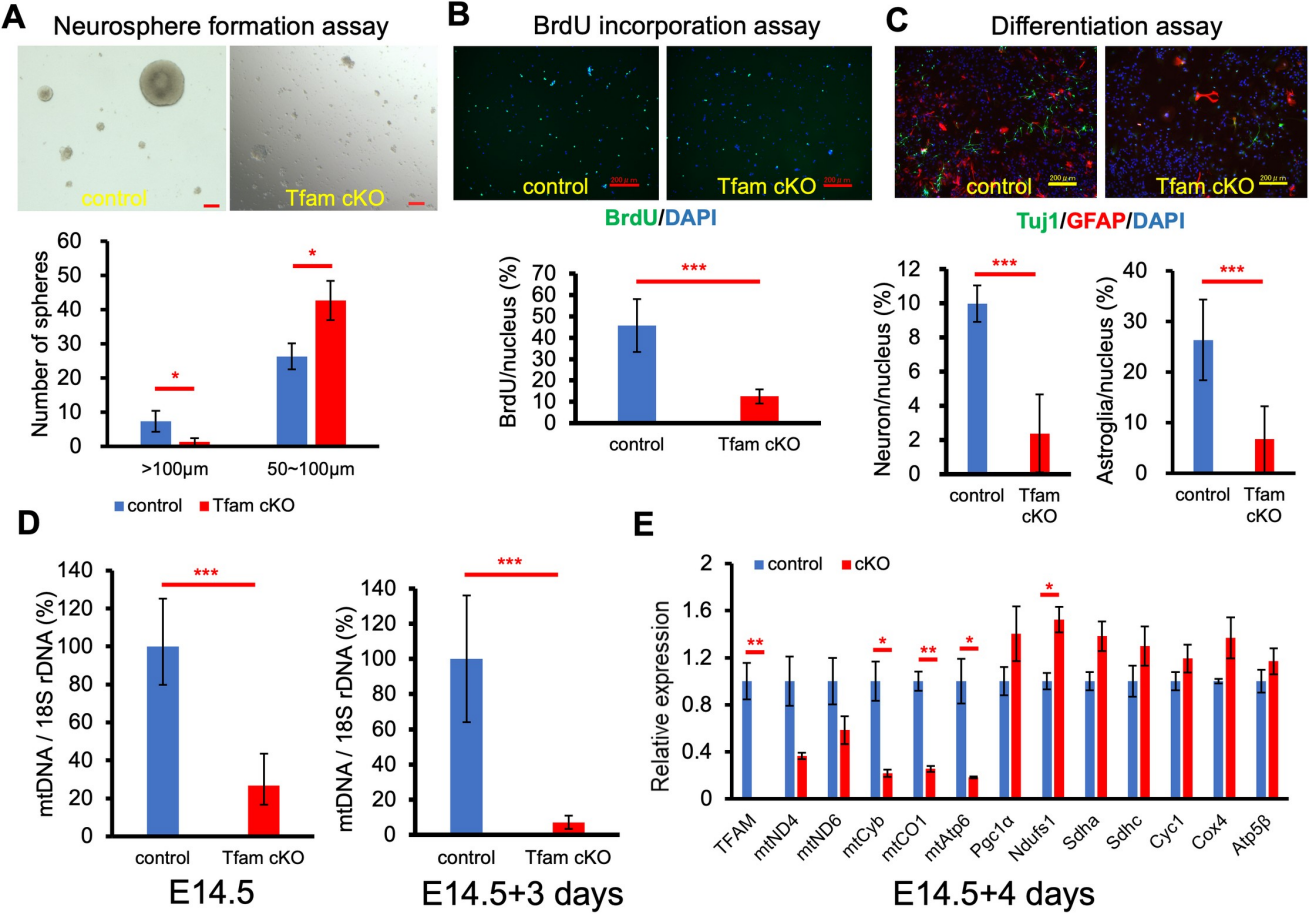
Neurosphere analysis. (A) Neurosphere formation assay. Representative images are shown. The number and the size of the spheres were measured, and the distribution of sphere number based on sphere size is shown. At least three independent clones per genotype were measured. Scale bars, 100 μm. Graph shows means ± SD. *P < 0.05. (B) BrdU incorporation assay. Cultured NSCs were incubated with 10 μM BrdU for 2 h and incorporated BrdU was detected using an anti-BrdU antibody. BrdU positive cells were counted. Graph shows means ± SD. ***P < 0.001. At least two independent clones per genotype were measured. Scale bars, 200 μm. (C) Differentiation of NSCs. NSCs on coverslips were cultured in differentiation medium for 7 days. Neurons and astroglia were detected by immunostaining with anti-Tuj1 and anti-GFAP antibodies, respectively. DAPI was used for nuclear staining. Graph shows means ± SD. ***P < 0.001. At least three independent clones per genotype were measured. Scale bars, 200 μm. (D) The relative mtDNA copy number analysis. The amount of mtDNA was measured by qRT-PCR using the 18S rRNA gene for normalization. Graph shows the control as 100%. DNA in the left panel was isolated from cortex cells at E14.5 and DNA in the right panel were isolated from 3-day cultured neurospheres. Graph shows means ± SD. n = 3 per group. ***P < 0.001. (E) Relative expression analysis of mtDNA- and nDNA-encoded OXPHOS genes. The expression levels of each gene were normalized with the expression levels of β-actin. Graph shows means ± SD. n = 3 per group. *P < 0.05; **P < 0.01.

To further elucidate the role of TFAM in neurogenesis, we conducted studies in vitro using NSCs and NPCs using a neurosphere assay. A single cell suspension was prepared from the forebrains of E14.5 mice, cultured for 4–6 days in NPC growth medium to form neurospheres, and then the amplified cells were used for further studies. In order to evaluate cell proliferation, we seeded the cells and cultured them for 10 days and the number and size of the neurospheres were counted. The Tfam cKO cells exhibited a reduced ability to form large colonies compared with control cells (**Fig 2A**).

To evaluate the proliferation of cultured NSCs, we used BrdU incorporation followed by the detection of BrdU-positive cells with an anti-BrdU antibody. The percentage of BrdU positive cells in the control was approximately 40 compared with 10 in the Tfam cKO group (**Fig 2B**). This indicated that the proliferation of NSCs from Tfam cKO was significantly less compared with that of the controls.

We next examined the differentiation ability of NSCs from Tfam cKO to maintain cells in differentiation medium for 7 days. Following differentiation, the neurons and astroglia were identified by Tuj1 and GFAP immunostaining, respectively. As shown in **Fig 2C**, there were fewer Tuj1-positive neuronal cells from the Tfam cKO group compared with the controls. The results of this in vitro study were consistent with that of in vivo studies and suggested that reduced Tfam function affects the neural differentiation of NSCs during neurogenesis. The relative number of astroglial cells was also reduced in the Tfam cKO group. However, because those cells were weakly stained with anti-GFAP antibody and were morphologically similar to astroglia cells in the Tfam cKO population, the expression of GFAP in astroglia from Tfam cKO cells may be retarded or weakened.

The relative amount of mtDNA in the cortical cells from brains at E14.5 was compared with that in cells cultured in NSC growth medium for 3 days after derivation at E14.5 (E14.5+3). The relative amount of mtDNA in Tfam cKO cells was approximately 20% of that in the control cells, whereas it was less than 10% in the Tfam cKO cells compared with the controls at E14.5+3 (**Fig 2D**). These results suggest that the decrease in mtDNA with Tfam deficiency progresses in a time-dependent manner and the relative amount of mtDNA in Tfam cKO cells at E14.5+3 is equivalent to that of the P0 brain (**Figs 1E and 2D**).

The expression of genes encoded on mtDNA and nuclear DNA (nDNA) from NSCs cultured for 4 days in NSC growth medium was examined. We designed a primer pair for the floxed exon of the Tfam gene. Only Tfam transcripts produced from wild-type alleles were detectable using these primers. We first examined TFAM expression in Tfam cKO cultured NSCs. The expression of Tfam mRNA was minimally detectable in Tfam cKO cells. The expression of genes encoded by nDNA relating to bioenergetics were upregulated in Tfam cKO cells compared with that of control cells, probably as a result of compensation. In contrast, the expression of genes encoded by mtDNA was significantly decreased in Tfam cKO cells compared with that of control cells (**Fig 2E**).

### Mitochondrial function is modified in Tfam cKO cells

**Fig 3:**
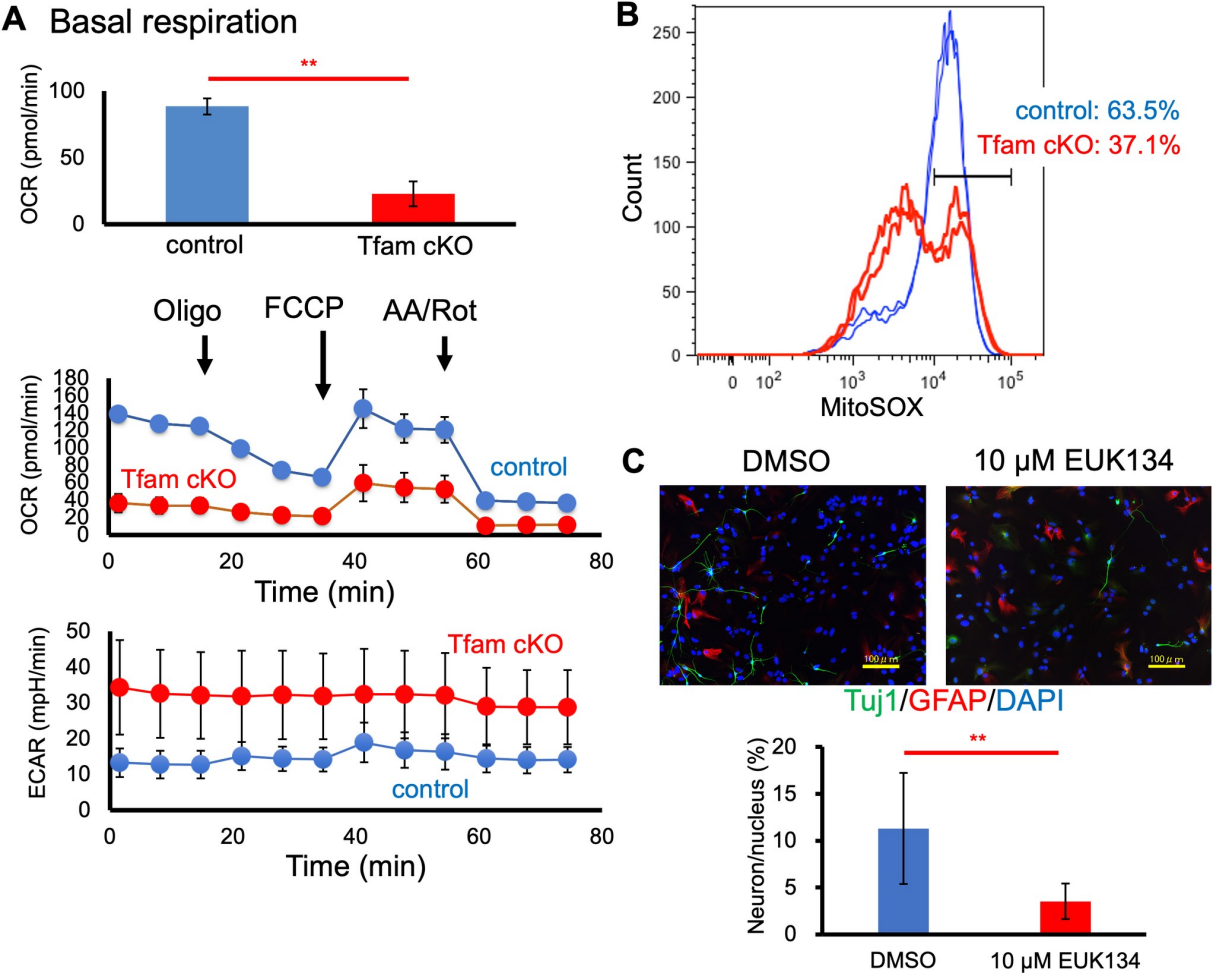
Altered cellular metabolism and ROS production in Tfam cKO cultured NSCs. (A) Oxygen consumption rate (OCR) and extracellular acidification rate (ECAR) of control and Tfam cKO NSCs were measured using a flux analyzer. Basal respiration, OCR, and ECAR are indicated. The graph shows means ± SD. n = 3 per group. **P < 0.01. At least three independent clones per genotype were measured. (B) Mitochondrial ROS was measured by flow cytometry using the ROS-sensitive MitoSOX Red dye. The results from two controls (blue) and two Tfam cKO NSCs (red) are shown. Two independent experiments were performed. (C) Immunocytochemical analysis of differentiation of control NSCs cultured in the presence of either EUK134 or DMSO as a vehicle control. Neurons and astroglia were detected by immunostaining using anti-Tuj1 and anti-GFAP antibodies. DAPI was used for nuclear staining. The graph shows means ± SD. **P < 0.01. Two independent experiments were performed. Scale bars, 100 μm.

To study how decreased Tfam in cells influences metabolism, we conducted a Mito-stress test using ETC complex inhibitors with an extracellular flux analyzer (**Fig 3A**). We used NSCs cultured for 4 days in NSC growth medium for the assay. As expected, Tfam cKO cells exhibited a significantly lower OCR compared with that of controls throughout the course of the Mito-stress test. The basal OCR of Tfam cKO cells was decreased more than 3-fold compared with that of control cells. The higher extracellular acidification rate (ECAR) of Tfam cKO cells compared with controls suggests that lactate production was induced in Tfam cKO cells. This result indicates that oxidative phosphorylation (OXPHOS) was suppressed, whereas glycolysis was enhanced as compensation in Tfam cKO cells.

Because ROS may be involved in neural differentiation, we used flow cytometry to analyze ROS levels in cells stained with MitoSox, an indicator of superoxide (O₂^·^⁻) of mitochondrial origin. As shown in **Fig 3B,** suppression of mitochondrial activity resulting from Tfam deficiency led to low production of superoxide from mitochondria (MitoSOX high population: 37.1% in Tfam cKO vs 63.5% in controls). This suggests inefficient oxygen consumption in Tfam cKO cells caused reduced ROS production. To test whether reduced ROS is related to impairment in neural differentiation of Tfam cKO NSCs, we treated control NSCs with EUK134, a superoxide scavenger [35, 36], during neuronal differentiation. EUK134 treatment decreased neuronal differentiation, but not glial differentiation (**Fig 3C**). This suggests that a specific amount of ROS may be required for efficient neural differentiation and decreased ROS production may be responsible for neural differentiation deficiency in Tfam cKO cells.

### Morphological abnormality of mitochondria in neurons and reduced expression of components of the electron transport chain are observed in Tfam cKO brains

**Fig 4:**
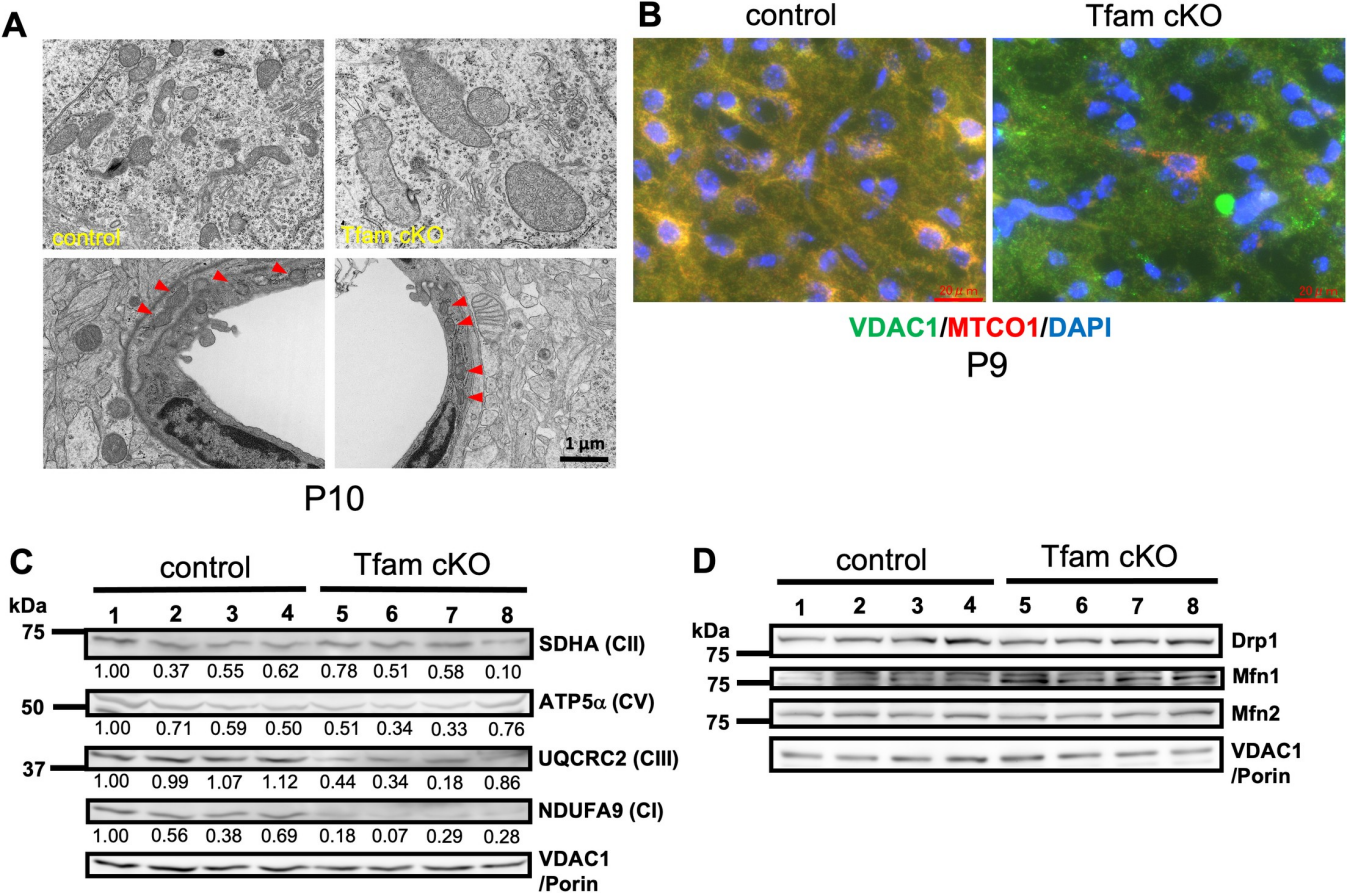
Mitochondrial defects in Tfam cKO mouse brains. (A) Ultrastructural analysis of mitochondria by transmission electron microscopy (TEM). Representative TEM images of brain sections from control and Tfam cKO at P10. In Tfam cKO brains, mitochondria in pyramidal cells in layer V shown in the upper panels are swollen and enlarged. On the other hand, mitochondria in endothelial cells (indicated by arrowheads) shown in the lower panels appear normal. At least two independent brains per genotype were examined. Scale bar, 1 μm. (B) Immunofluorescence of VDAC1/Porin and MTCO1 with DAPI nuclear staining for pyramidal cells in layer V at P9. Scale bars, 20 μm. (C) Western blot analysis of protein levels in brains from Tfam cKO and control at P8. Samples were probed with antibodies against SDHA, ATP5α, UQCRC2, NDUFA9, and VDAC1/Porin. (D) Western blot analysis of protein levels of mitochondrial fission and fusion related proteins in brains from Tfam cKO and control at P8. Samples were probed with antibodies against Drp1, Mfn1, Mfn2, and VDAC1/Porin.

To evaluate mitochondrial morphology in Tfam cKO brains, pyramidal cells from the brain cortex at P10 were examined by transmission electron microscopy (TEM). We found that mitochondria in the pyramidal cells of Tfam cKO were enlarged and swollen with rough cristae compared with those of the controls (**Fig 4A**). In contrast, mitochondria in the perivascular cells of Tfam cKO appeared similar to those of the controls (**Fig 4A**). This indicated that conditional deletion of TFAM driven by the nestin promoter prominently affects neurons derived from NSCs (**Fig 4A**). We further confirmed a loss of mtDNA-encoded gene expression by immunohistochemistry in P9 brains using antibodies against VDAC1/Porin and MTCO1. Although VDAC1/Porin staining in Tfam cKO brain was comparable with that in control brain, MTCO1 positive neurons at comparable levels with control were significantly reduced (< 10% compared with control) in the Tfam cKO brain (**Fig 4B**).

We next examined protein expression levels of ETC components in Tfam cKO brains at P8 by western blot analysis. Expression of NDUFA9 (complex I) and UQCRC2 (complex III) in Tfam cKO brains was significantly decreased compared with that of control brains and expression of VDAC1/Porin, which was used as a loading control for mitochondria, was similar. Expression of SDHA composing complex II in Tfam cKO brains was similar or slightly higher compared with that in the control brains. Because complex I and III are composed of both mtDNA- and nDNA-encoded subunits, the reduction of mtDNA-encoded subunits may cause the complexes to be unstable. This may explain why SDHA, a subunit of complex II which does not include a subunit encoded in mtDNA, was not affected in Tfam cKO (**Fig 4C**). We examined the expression of several mtDNA-encoded mRNAs using brain RNA at P0, P4, and P8 by qRT-PCR (S1 Fig). The expression levels of some mtDNA-encoded mRNAs were already less than 20% of the controls even at P0. Mitochondrial activity was most likely dependent on the mtDNA-encoded proteins rather than the levels of mtDNA-encoded mRNAs. The expression of nDNA-encoded ETC related proteins in Tfam cKO brains at P0 was maintained at adequate levels compared with those in control brains (S2 Fig). This suggested that enough ETC activity is still maintained in Tfam cKO brain at P0. Although we tried to detect COI and COII, which are mtDNA-encoded proteins, by WB using commercially available antibodies, we could not detect any specific bands and could not evaluate their expression by WB. These results indicated that the TFAM defect caused mitochondrial impairment in Tfam cKO brains.

Because mitochondria in the neuronal cells of Tfam cKO were enlarged, we next examined protein expression levels of mitochondrial fission and fusion related proteins in Tfam cKO brains at P8 by western blot analysis (Fig 4D). The expression levels of both fission related Drp1 and fusion related Mfn2 in Tfam cKO brains were comparable with those in control. The expression of another fusion related Mfn1 was slightly upregulated in Tfam cKO brains compared with that in control. These results indicated that mitochondrial enlargement in Tfam cKO brains is most likely caused by hyperfusion of mitochondria rather than reduction of mitochondrial fission activity. Similar to our observation, West et al. also showed that TFAM knockdown (KD) in mouse embryonic fibroblasts (MEFs) caused mitochondrial hyperfusion [37]. They also showed that this TFAM KD induced mitochondrial hyperfusion was rescued by KD of Mfn1, another mitochondrial fusion related protein.

### Apoptosis does not occur at early periods of neurogenesis and precedes the activation of microglia in Tfam cKO brain cortexes

**Fig 5:**
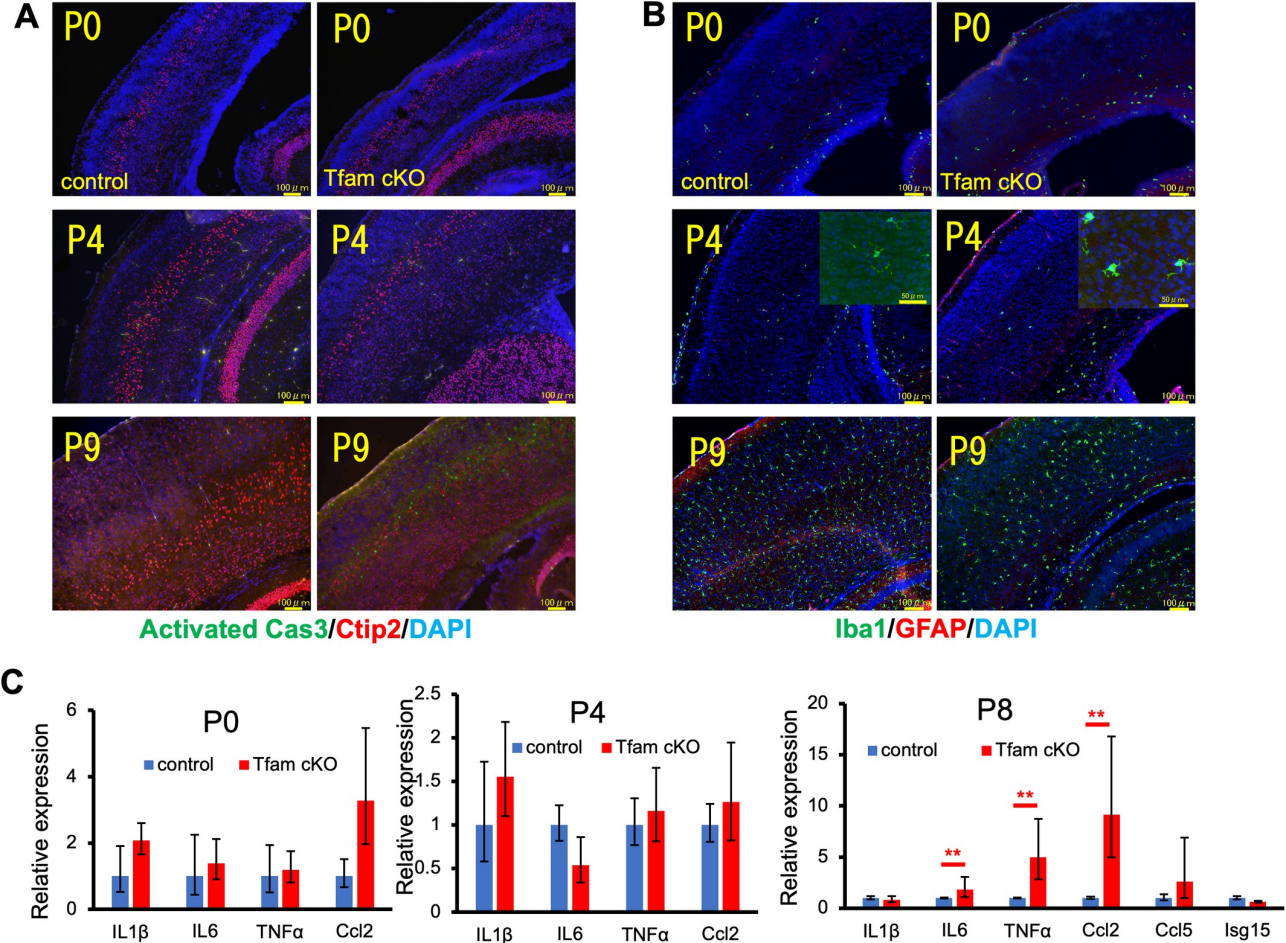
Apoptotic cell death and microglial activation in Tfam cKO brains. (A) Representative images of immunostaining with antibodies against activated caspase 3 and Ctip2 with DAPI nuclear staining of brains at P0, P4, and P9. Activated caspase 3-positive apoptotic cells can be detected in layer V neurons in Tfam cKO brains at P9. Scale bars, 100 μm. (B) Representative images of immunostaining with antibodies against Iba1 and GFAP with DAPI nuclear staining of brains at P0, P4, and P9. Iba1-positive activated microglia are detected in Tfam cKO brains at P4. Scale bars, 100 μm. (C) The expression of inflammatory genes by qRT-PCR. The expression levels of some inflammatory genes in Tfam cKO brains at P8 were significantly upregulated compared with those in control brains. Graph shows means ± SD. n = 3 mice per group. **P < 0.01.

We hypothesize that apoptosis may be involved in the thinning brain cortex in Tfam cKO. To test this possibility, we performed immunohistochemistry of brain cortexes at P0, P4, and P9 with antibodies against activated caspase 3 (cas3), an apoptosis marker, and Ctip2, a marker of layer 5 in neocortex. Apoptosis occurred primarily in pyramidal neurons at layer 5 which was identified by Ctip2 immunostaining in Tfam cKO, but not in the controls at P9. However, apoptotic cells were barely detectable in the brain of Tfam cKO or the controls at P0 and P4 (**Fig 5A**). Thus, apoptosis may not be a major contributor of brain microcephaly specific to Tfam cKO.

Microglia are involved in brain homeostasis by contributing to various physiological and pathophysiological processes. To evaluate microglial status in the Tfam cKO brain, we evaluated microglia by immunostaining with an antibody against Iba1, which is a marker for microglia. Activated microglia, which can be recognized by transformation from a ramified to an ameboid morphology, were observed throughout the cortex in Tfam cKO brains at P9. Although activated microglia were hardly detectable in P0 brain, they were observed in Tfam cKO brains, but not in control brain at P4. It is noteworthy that activation of microglia precedes apoptosis in neuronal cells (**Fig 5B**).

To investigate the expression of genes which are related to inflammation and activation of microglia in the cortex, we selected IL-1β, IL-6, TNFα, Ccl2, Ccl5, and Isg15 as targets for qRT-PCR analysis. Expression of the inflammatory genes, IL-6, TNFα, and Ccl2, was increased in Tfam cKO brains at P8 compared with that in the control brains, but not at P0 and P4 (**Fig 5C**). Expression of the downstream targets of the cGas-stimulator of interferon genes (STING) pathway including IL-1β, Ccl5, and Isg15 [38] were not affected in Tfam cKO brains. These results suggest that the cGAS-STING pathway was not activated in Tfam cKO brains and upregulation of inflammatory cytokines, such as IL-6 and TNFα, primarily reflect activation of microglia in Tfam-deficient brains.

### Integrated stress response is induced *in vitro* and *in vivo*

**Fig 6:**
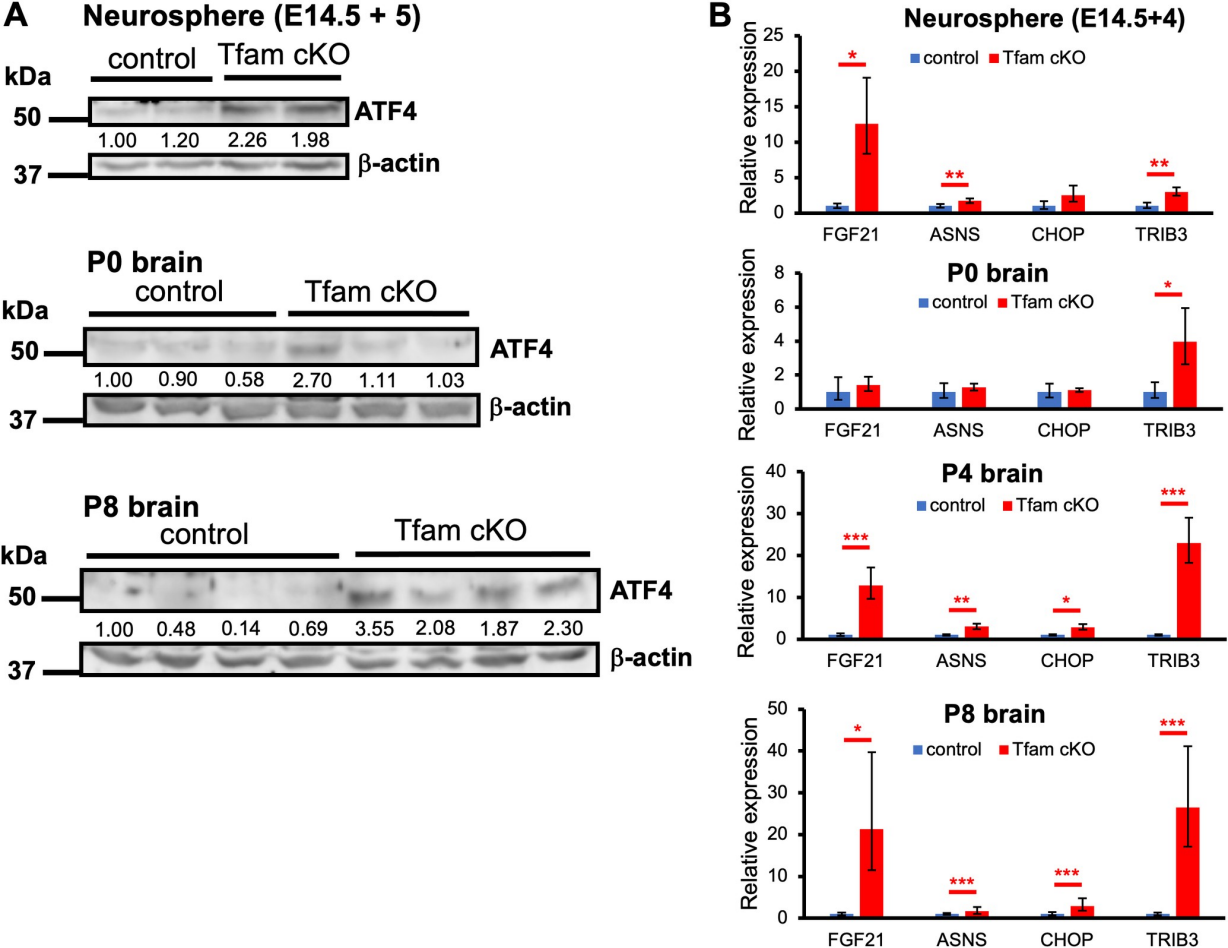
Activation of ISR in Tfam cKO brains and cultured NSCs. (A) Western blot analysis of ATF4 for cultured neurospheres (E14.5 + 5), P0, and P8 brains. Beta-actin was used as a loading control. (B) The expression of ATF4 target genes by qRT-PCR. Total RNA was isolated from neurospheres (E14.5 + 4), P0, P4, and P8 brains. The expression levels of each gene were normalized using β-actin. Graph shows means ± SD. n = 3 mice per group. *P<0.05; **P < 0.01; ***P < 0.001.

ISR can be induced by mitochondrial dysfunction through ATF4 activation in vitro. We first performed western blot analysis to detect ATF4 in brains at E14.5, P0, and P8 (**Fig 6A**). We confirmed that ATF4 accumulated in Tfam cKO brains at P8 as well as in cultured neurospheres from Tfam cKO brains. The trend of ATF4 accumulation in P0 brains was evident. To further investigate whether ISR was activated, we examined the expression of ATF4 target genes in Tfam cKO brains. As shown in **Fig 6B**, expression of ATF4 target genes was significantly upregulated in Tfam cKO brains at P4 and P8 compared with that in control brains as well as cultured neurospheres. A similar trend was observed in P0 brains. This indicates that mitochondrial disfunction induced by loss of Tfam through reduction of mtDNA, instability of ETC, energy shortage, and reduction of ROS production, may cause ATF4 accumulation and induce ISR.

## Discussion

In this study, we addressed the role of mitochondrial function and activity on neurogenesis using a neural stem cell-specific Tfam inactivation mouse model. TFAM is an essential factor for maintaining and replicating mtDNA. Inactivation of Tfam genes in NSCs causes a copy number reduction of mtDNA in mouse brains and results in impairment of OXPHOS because mtDNA-encoded subunits of OXPHOS decrease rapidly. The copy number of mtDNA in Tfam cKO brains at P0 is already less than 10% compared with those in control brains. Cultured NSCs from Tfam cKO brains exhibit weak OCR because of a reduction in mtDNA. As a result, they show defects in cell growth and differentiation into neurons. Reduced mitochondrial activity through the impairment of OXPHOS in NSCs during neurogenesis causes activation of the ISR pathway through the accumulation of the ATF4 transcription factor to maintain brain function.

Although the copy number of mtDNA in Tfam cKO brains is already significantly reduced at P0, Tfam cKO neonates appear normal with no significant differences among control littermates. Brain histology is also indistinguishable at P0 and the abnormality in the Tfam cKO brains starts to appear after P4. The nestin promoter is active in NSCs after E11.5 and all lineages derived from NSCs may be affected by Tfam inactivation. Although we expected that an abnormality of Tfam cKO brains would have appeared during neurogenesis at embryonic stages and the role of mitochondrial activity in NSCs during neurogenesis in vivo would have become clear, we could not resolve this issue using the mouse model at this stage. Similar to other mouse models of Tfam cKO in the heart [39, 40], skeletal muscle [39, 41], pancreatic cells [42], and cortical neurons [25], there may be a time lag until phenotypes resulting from Tfam inactivation occur in NSCs. This may depend on the stability of mitochondrial transcripts or mtDNA-encoded subunits of the respiratory chain because the expression of transcripts encoded in nDNA was not affected by Tfam inactivation.

Recently, Bartesaghi et al. showed that inhibition of respiration by knockdown of the complex I subunit NDUFA10 in cultured NSCs resulted in a metabolic shift to a more glycolytic state [43]. This shift in NSCs has a significant advantage for cell growth. Although we did not observe clear differences in brains at P0 between Tfam cKO and the control, a metabolic shift may occur in NSCs with Tfam inactivation and compensate for disadvantages in mitochondrial impairment in NSCs from Tfam cKO mice. Stem cells are generally considered to be glycolytic [44] and metabolic shift from glycolytic to aerobic metabolism which is relied on oxidative phosphorylation (OXPHOS) in mitochondria occurs during neurogenesis [45–47]. This metabolic shift is accompanied with increased mitochondrial biogenesis. TFAM is an essential factor for mitochondrial biogenesis through maintenance of the mtDNA copy number. Impairment of TFAM function causes decreased mitochondrial biogenesis and mitochondrial dysfunction and metabolic shift might not occur effectively during neurogenesis in Tfam cKO brain. Inefficient mitochondrial biogenesis during neurogenesis might induce mitochondrial stress and this most likely cause activation of ISR following ATF4 accumulation to maintain normal function.

Augustyniak et al. have reported that mitochondrial biogenesis influences neural fate commitment in the developmental stage dependent manner. They established in vitro differentiated neural stem cells (NSC), early neural progenitors (eNP), and neural progenitors (NP) from human induced pluripotent stem cells (hiPSC) and examined the sensitivity to the factors involved in mitochondrial biogenesis for these cells. Interestingly, pyrroloquinoline quinone (PQQ) which positively affects the mitochondrial biogenesis and idebenone (IDB), the synthetic analog of coenzyme Q10 specifically enhanced mitochondrial biogenesis in eNP [48, 49]. On the other hand, enhanced mitochondrial biogenesis by bezafibrate (BZ) which upregulates PGC-1α expression through activation of peroxisome proliferator-activated receptors (PPARs) was most effective for NP [50]. Fate commitment decision was also differentially affected by each compound and activation of mitochondrial biogenesis might affect the final outcome in neurogenesis. Our observation also indicated that improper maintenance of mitochondrial biogenesis during neurogenesis largely affects for construction of functional brain.

The cyclic GMP-AMP synthase (cGAS) and its downstream effector pathway, STING, activates the innate immune response in response to viral infection [51]. Mitochondrial stress causes mtDNA release from mitochondria and released mtDNA acts as danger signal and stimulates the cGAS-STING pathway to activate the innate immune response [52]. West et al. recently showed that mtDNA is released from mitochondria and the cGAS-STING pathway is activated in heterozygous Tfam mouse embryonic fibroblasts (Tfam^+/-^ MEFs), in which moderate mtDNA stress is observed [37, 53]. The expression of interferon-stimulating genes (ISGs) and antiviral signaling factors is activated in Tfam^+/-^ MEFs. The expression of ISGs, such as Isg15, is not activated in Tfam cKO (Fig 5C) and Tfam heterozygous (Tfam^F/+^; Nestin-Cre^+^, data not shown) brains or cultured NSCs. Activation of the mtDNA stress response through the cGAS-STING pathway may be cell type- or tissue-dependent. Microglia is activated in Tfam cKO brains prior to apoptosis. Similar to results showing impairment of autophagy by FIP200 inactivation in NSCs during neurogenesis [54], the expression of Ccl5 in Tfam cKO brains is upregulated and microglia may exhibit accelerated migration and activation by secreted factors from Tfam-deficient cells. These findings warrant further study.

ISR is an adaptive pathway that leads to global translation arrest and specific gene activation in response to environmental stresses and pathological conditions [55, 56]. The key factor in this pathway is the ATF4 transcription factor. Translation of ATF4 mRNA is activated through the phosphorylation of eukaryotic translation initiation factor 2 alpha (eIF2α) and ATF4 accumulates and promotes cellular recovery. Mitochondrial stresses, such as disruption of mitochondrial protein homeostasis and impairment of mitochondrial electron transport chain, activate ISR to repair the mitochondria [57–59]. One of the four kinases, heme-regulated inhibitor (HRI), is activated in response to mitochondrial stress. Mitochondrial stress induces metalloendopeptidase OMA1-dependent cleavage of the DAP3-binding cell death enhancer 1 (DELE1) which exists in the mitochondrial matrix. Cleaved DELE1 is translocated into cytosol and activates HRI. Because Tfam inactivation in NSCs in neurogenesis causes impairment of OXPHOS because of mtDNA defects and Tfam-deficient cells cannot maintain proper mitochondrial function, HRI mediated ISR might be activated in this model. In fact, ATF4, ISR downstream target, accumulates in Tfam cKO brains and cultured NSCs, and ATF4 target genes such as FGF21 and TRIB3 are upregulated. It is believed that ISR is induced as an adaptive response. This indicates that ISR mediated by ATF4 is a central pathway that responds to mitochondrial impairment in cultured NSCs and brain during neurogenesis.

Similar to TFAM cKO, ISR is also activated in brain in which mitochondrial fission protein Drp1 inducibly deleted by CaMKIIa-CreERT2 [60]. Activation of ISR in Drp1 knockout neurons leaded to neuron-specific expression of FGF21. The authors suggested that the ER stress independent ISR pathway exists using chemical chaperone tauroursodeoxycholic acid (TUDCA) which reduces mitochondria-induced ER stress in this mouse model. As mentioned above, HRI mediated ISR pathway might act as mitochondrial stress-induced ISR pathway in this model as well as TFAM cKO. Mitochondrial dysfunction is associated with increased expression and secretion of FGF21 in many cell types [61–64]. Therefore, FGF21 might be a useful biomarker for neurodegenerative conditions caused by mitochondrial dysfunction.

In summary, our data demonstrate that TFAM is required for maintaining NSC function and the loss of TFAM in NSCs causes defects in brain formation by impairing neurogenesis. Our study also clarified the importance of the ATF4-mediated ISR pathway in cultured NSCs and brain on promoting cellular recovery from mitochondrial impairment induced by Tfam inactivation. These findings provide new insight for further investigation into the role of mitochondrial health and function in neurogenesis.

## Supporting information

S1 Fig Relative expression analysis of mtDNA- and nDNA-encoded OXPHOS genes in brains at P0, P4, and P8. The expression levels of each gene were normalized with the expression levels of β-actin. Graph shows means ± SD. n = 3 per group. *P < 0.05; **P < 0.01; ***P < 0.001.
(TIF)

S2 Fig Western blot analysis of protein levels in brains from Tfam cKO and control at P0. Samples were probed with antibodies against SDHA, UQCRC2, NDUFA9, and VDAC1/Porin.
(TIF)

S1 Table Primer sequences for relative mtDNA copy number analysis. (DOCX)

S2 Table Primer sequences for qRT-PCR analysis. (DOCX)

S1 Raw images (PDF)
