# Supplementary figures and images for: Loss of mitochondrial transcription factor A in neural stem cells leads to immature brain development and triggers the activation of the integral stress response in vivo
Source: PLoS One. 2021 Jul 28;16(7):e0255355. doi: 10.1371/journal.pone.0255355 (PMC8318236; doi:10.1371/journal.pone.0255355)

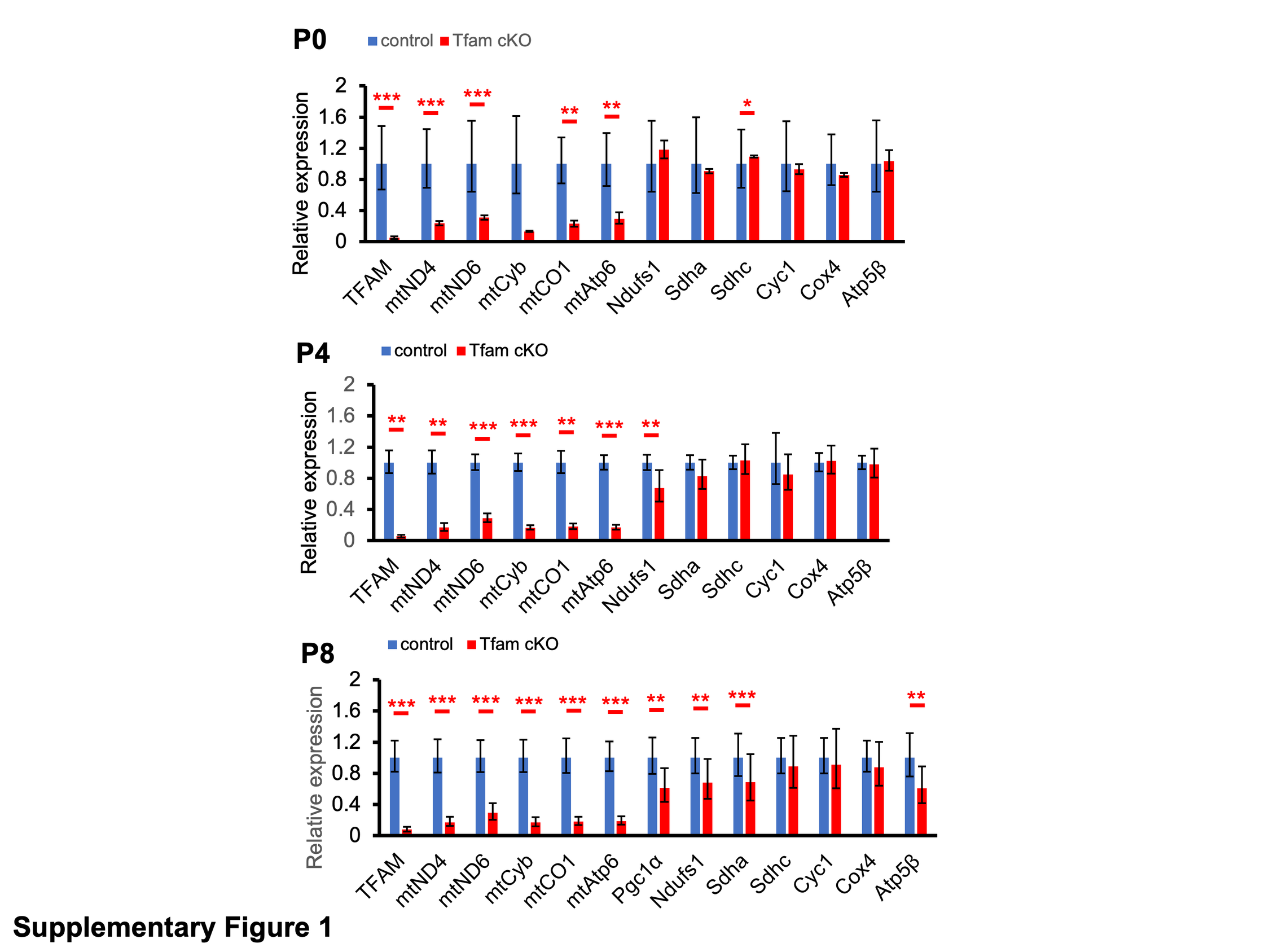

Supplement: S1 Fig — The expression levels of each gene were normalized with the expression levels of β-actin. Graph shows means ± SD. n = 3 per group. *P < 0.05; **P < 0.01; ***P < 0.001. (TIF) [file pone.0255355.s001.tif]

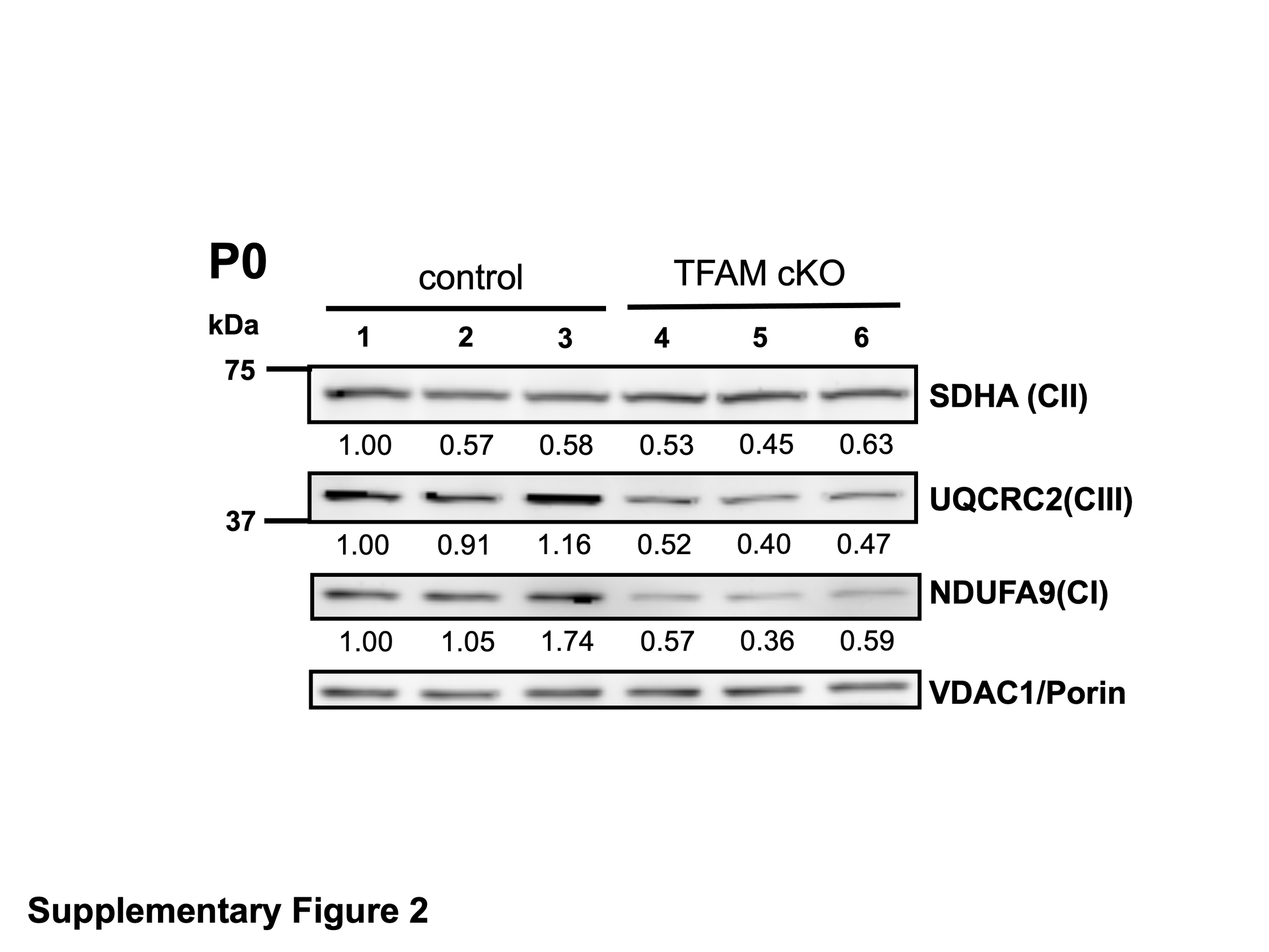

Supplement: S2 Fig — Samples were probed with antibodies against SDHA, UQCRC2, NDUFA9, and VDAC1/Porin. (TIF) [file pone.0255355.s002.tif]
