# Supplementary material for: Loss of mitochondrial transcription factor A in neural stem cells leads to immature brain development and triggers the activation of the integral stress response in vivo
Source: PLoS One. 2021 Jul 28;16(7):e0255355. doi: 10.1371/journal.pone.0255355 (PMC8318236; doi:10.1371/journal.pone.0255355)
Supplement: S1 Table — (DOCX) [file pone.0255355.s003.docx]

**S1 Table. Primer sequences for mtDNA copy number analysis**

| Target gene | Forward primer | Reverse primer |
| --- | --- | --- |
| mtDNA | TCCCAGACATACTAGGAGAC | ATTAAGGCTAGGACACCTCC |
| 18S rRNA | AGAGGGACAAGTGGCGTTC | CGCTGAGCCAGTCAGTGT |
