# Supplementary material for: Loss of mitochondrial transcription factor A in neural stem cells leads to immature brain development and triggers the activation of the integral stress response in vivo
Source: PLoS One. 2021 Jul 28;16(7):e0255355. doi: 10.1371/journal.pone.0255355 (PMC8318236; doi:10.1371/journal.pone.0255355)
Supplement: S2 Table — (DOCX) [file pone.0255355.s004.docx]

**S2 Table. Primer sequences for qRT-PCR analysis**

| Target gene | Forward primer | Reverse primer |
| --- | --- | --- |
| TFAM | CAAAGGATGATTCGGCTCAG | AAGCTGAATATATGCCTGCTTTTC |
| ND4 | CTAATAATCGCACATGGCCTC | CGTAGTTGGAGTTTGCTAGG |
| ND6 | AAAACGATCCACCAAACCCT | GGTTAGCATTAAAGCCTTCACC |
| Cyb | CCATTCTACGCTCAATCCCCA | AGGCTTCGTTGCTTTGAGGTA |
| CO1 | ACACAACTTTCTTTGATCCCG | AGAATCAGAACAGATGCTGG |
| Atp6 | CCTTCAATCCTATTCCCATCC | GTTGGAAAGAATGGAGACGG |
| Pgc1α | AGCCGTGACCACTGACAACGAG | GCTGCATGGTTCTGAGTGCTAAG |
| Ndufs1 | AGGATATGTTCGCACAACTGG | TCATGGTAACAGAATCGAGGGA |
| Sdha | GGAACACTCCAAAAACAGACCT | CCACCACTGGGTATTGAGTAGAA |
| Sdhc | GCTGCGTTCTTGCTGAGACA | ATCTCCTCCTTAGCTGTGGTT |
| Cyc1 | CAGCTTCCATTGCGGACAC | GGCACTCACGGCAGAATGAA |
| Cox4 | ATGTCACGATGCTGTCTGCC | GTGCCCCTGTTCATCTCGGC |
| Atp5b | GCAAGGCAGGGACAGCAGA | CCCAAGGTCTCAGGACCAACA |
| IL-1β | CTGTGACTCATGGGATGATGATG | CGGAGCCTGTAGTGCAGTTG |
| IL-6 | TAGTCCTTCCTACCCCAATTTCC | TTGGTCCTTAGCCACTCCTTC |
| TNFα | CCTGTAGCCCACGTCGTAG | GGGAGTAGACAAGGTACAACCC |
| Ccl2 | TTAAAAACCTGGATCGGAACCAA | GCATTAGCTTCAGATTTACGGGT |
| Ccl5 | GCTGCTTTGCCTACCTCTCC | TCGAGTGACAAACACGACTGC |
| Isg15 | GGTGTCCGTGACTAACTCCAT | TGGAAAGGGTAAGACCGTCCT |
| FGF21 | CTGCTGGGGGTCTACCAAG | CTGCGCCTACCACTGTTCC |
| Asns | CACAAGGCGCTACAGCAAC | CCAGCATACAGATGGTTTTCTCG |
| CHOP | CTGGAAGCCTGGTATGAGGAT | CAGGGTCAAGAGTAGTGAAGGT |
| Trib3 | GCAAAGCGGCTGATGTCTG | AGAGTCGTGGAATGGGTATCTG |
