# Supplementary material for: Loss of mitochondrial transcription factor A in neural stem cells leads to immature brain development and triggers the activation of the integral stress response in vivo
Source: PLoS One. 2021 Jul 28;16(7):e0255355. doi: 10.1371/journal.pone.0255355 (PMC8318236; doi:10.1371/journal.pone.0255355)

Figure 4C

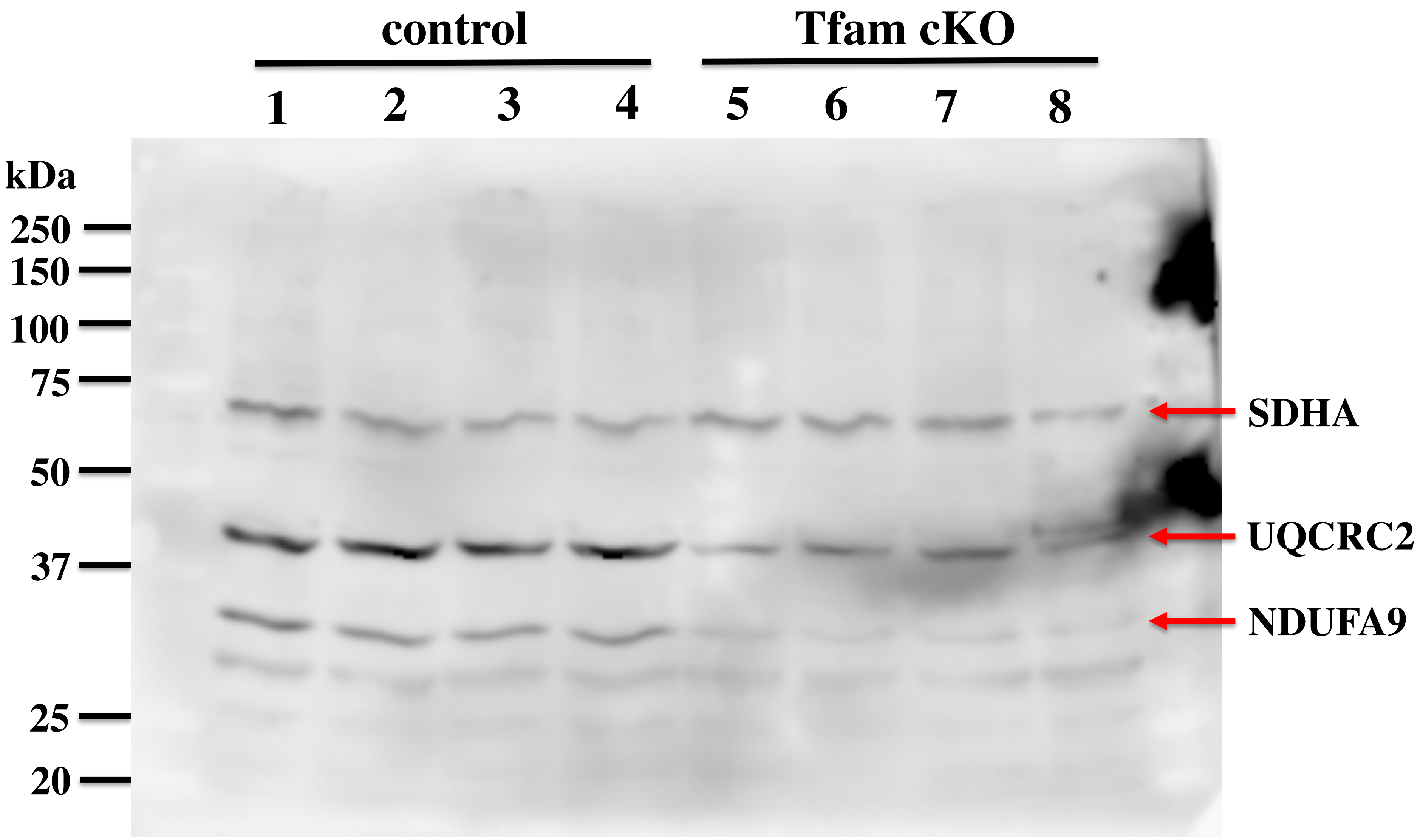
control
Tfam cKO
1
2
3
4
5
6
7
8
kDa
250
150
100
75
50
37
25
20
SDHA
UQCRC2
NDUFA9

Figure 4C

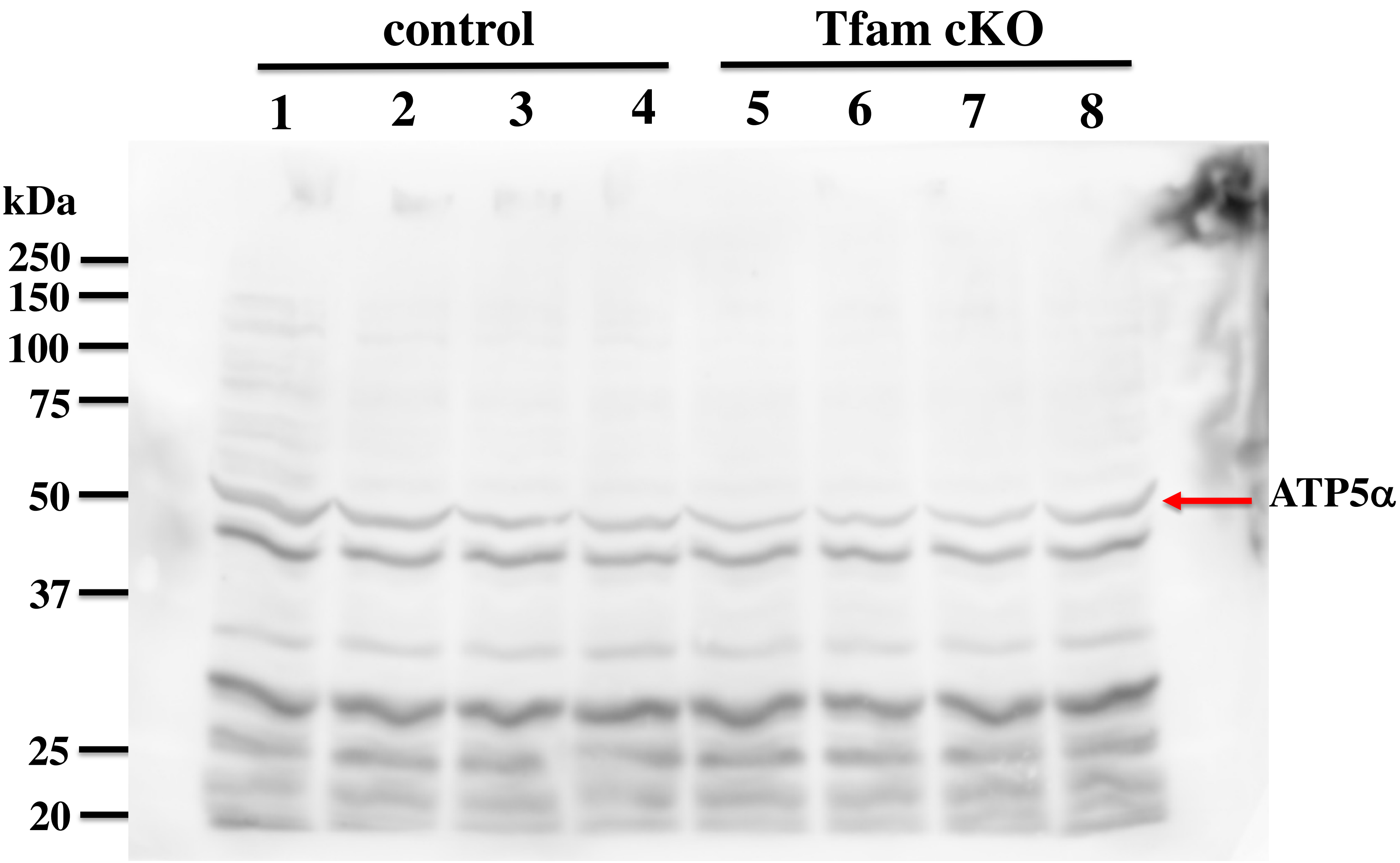

Figure 4C

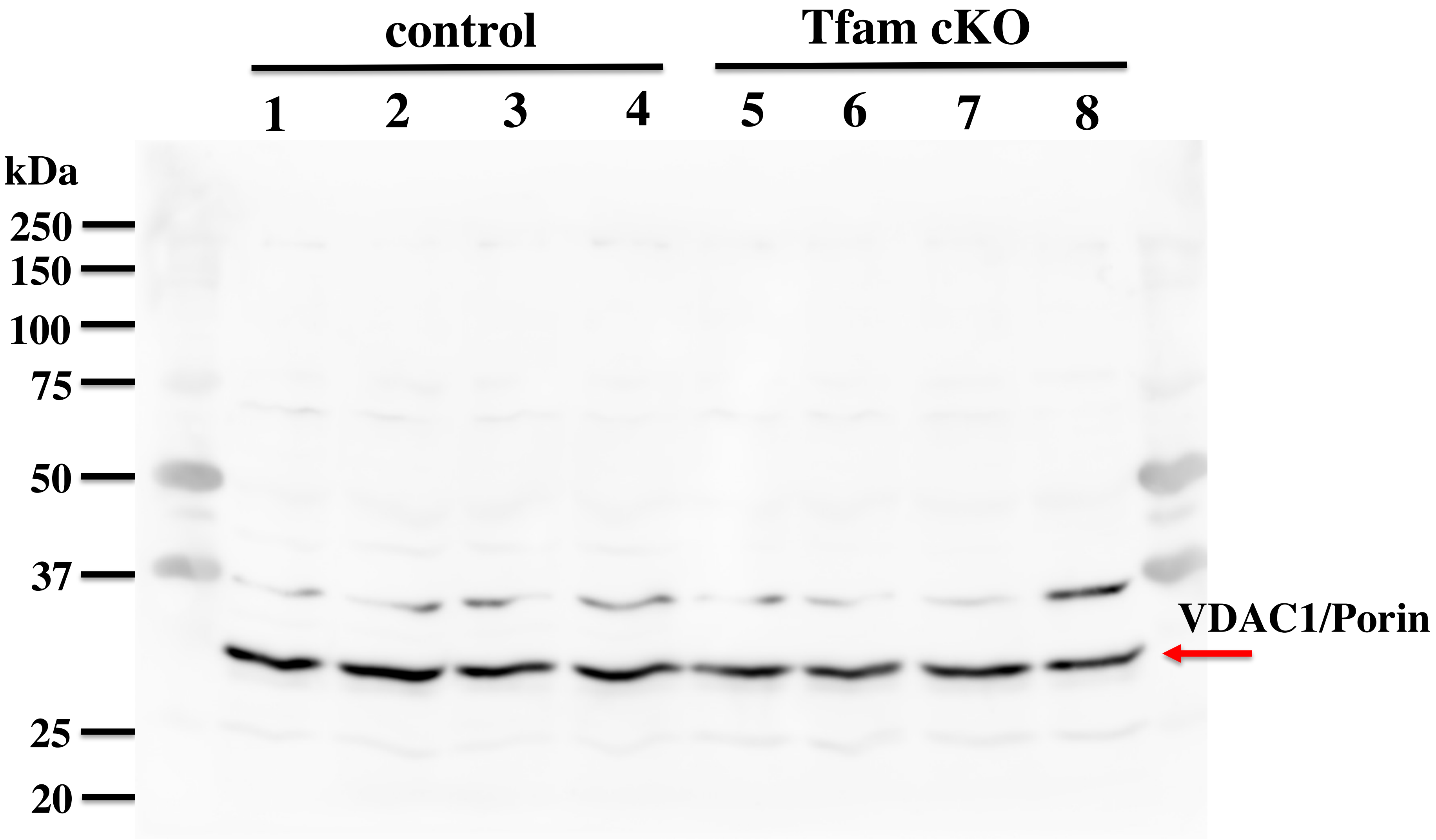
control
Tfam cKO
1
2
3
4
5
6
7
8
kDa
250
150
100
75
50
37
25
20
VDAC1/Porin

Figure 4D

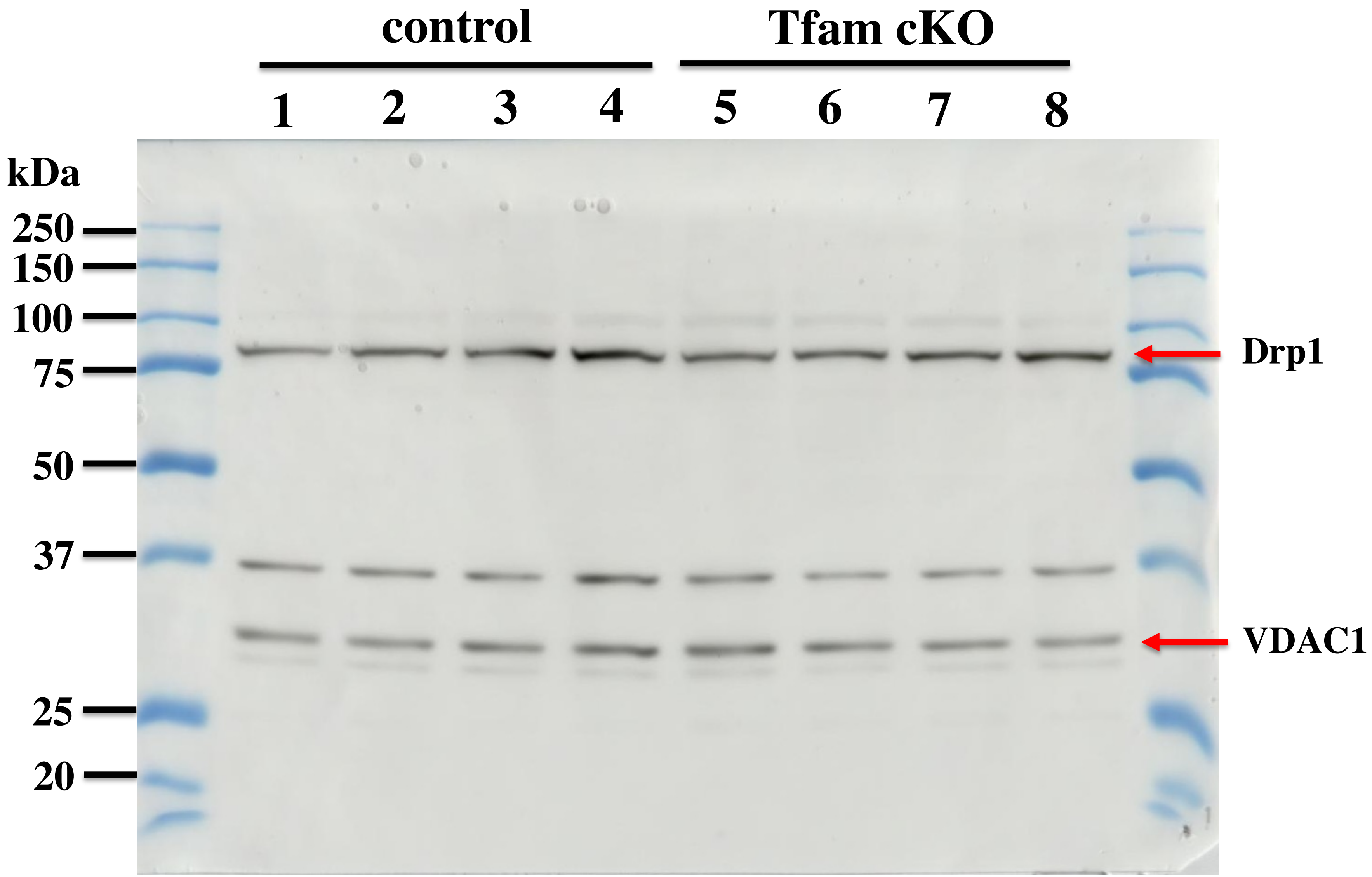

control
Tfam cKO
1
2
3
4
5
6
7
8
kDa
250
150
100
75
50
37
25
20
Drp1
VDAC1

Figure 4D

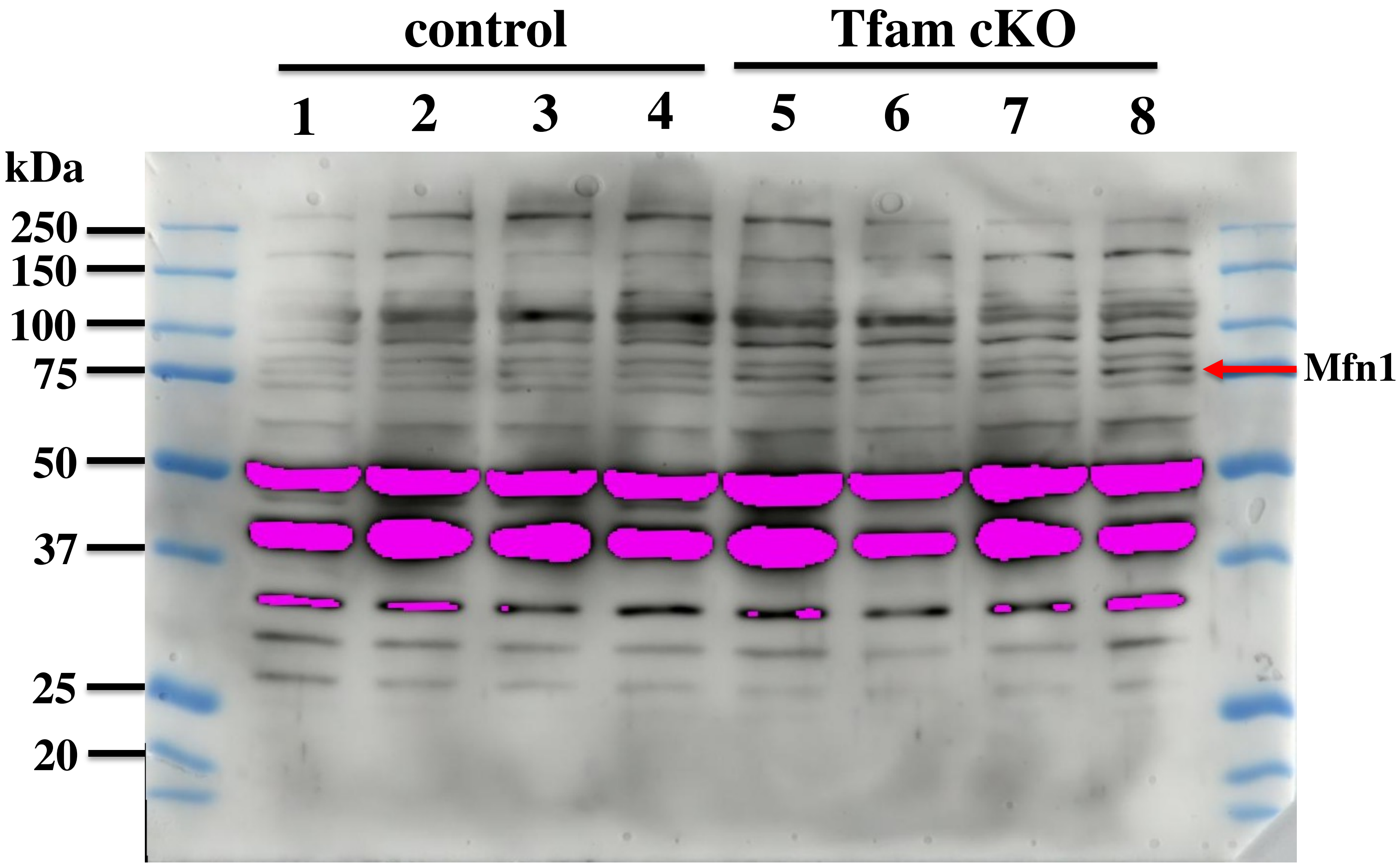

Figure 4D

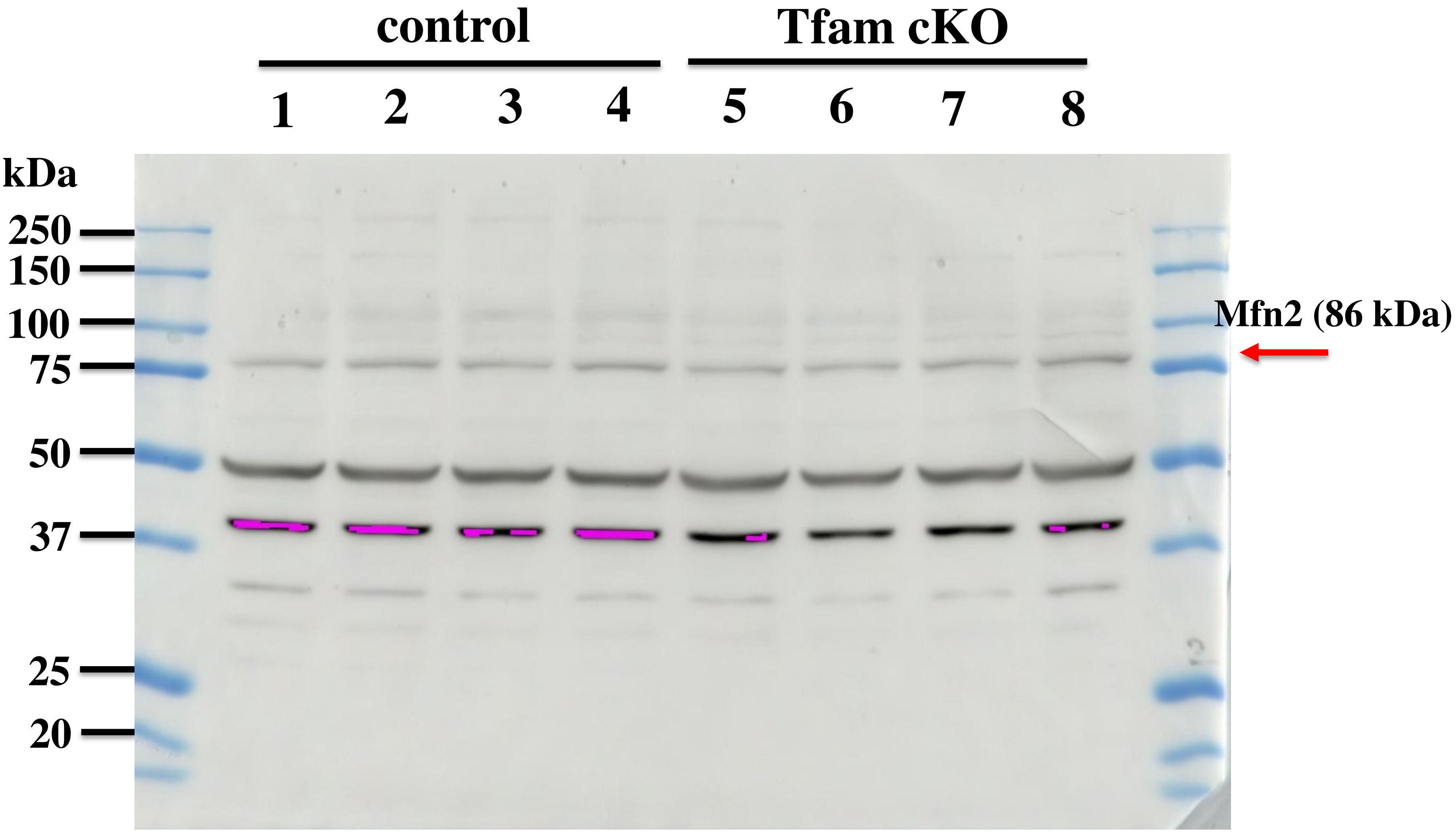

control
Tfam cKO
1
2
3
4
5
6
7
8
kDa
250
150
100
75
50
37
25
20
Mfn2 (86 kDa)

Figure 6A: Neurosphere (E14.5 + 5)

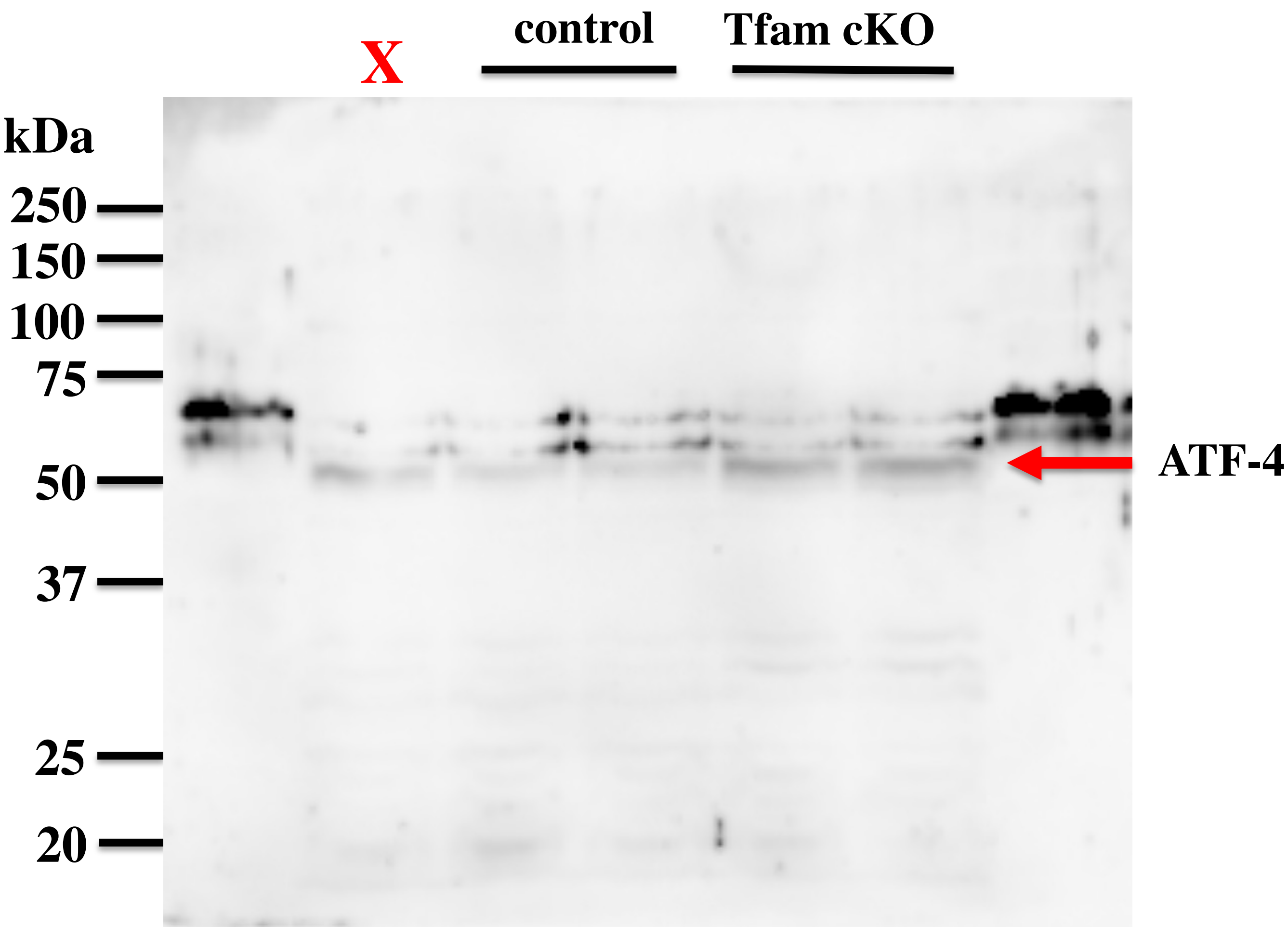

Figure 6A: Neurosphere (E14.5 + 5)

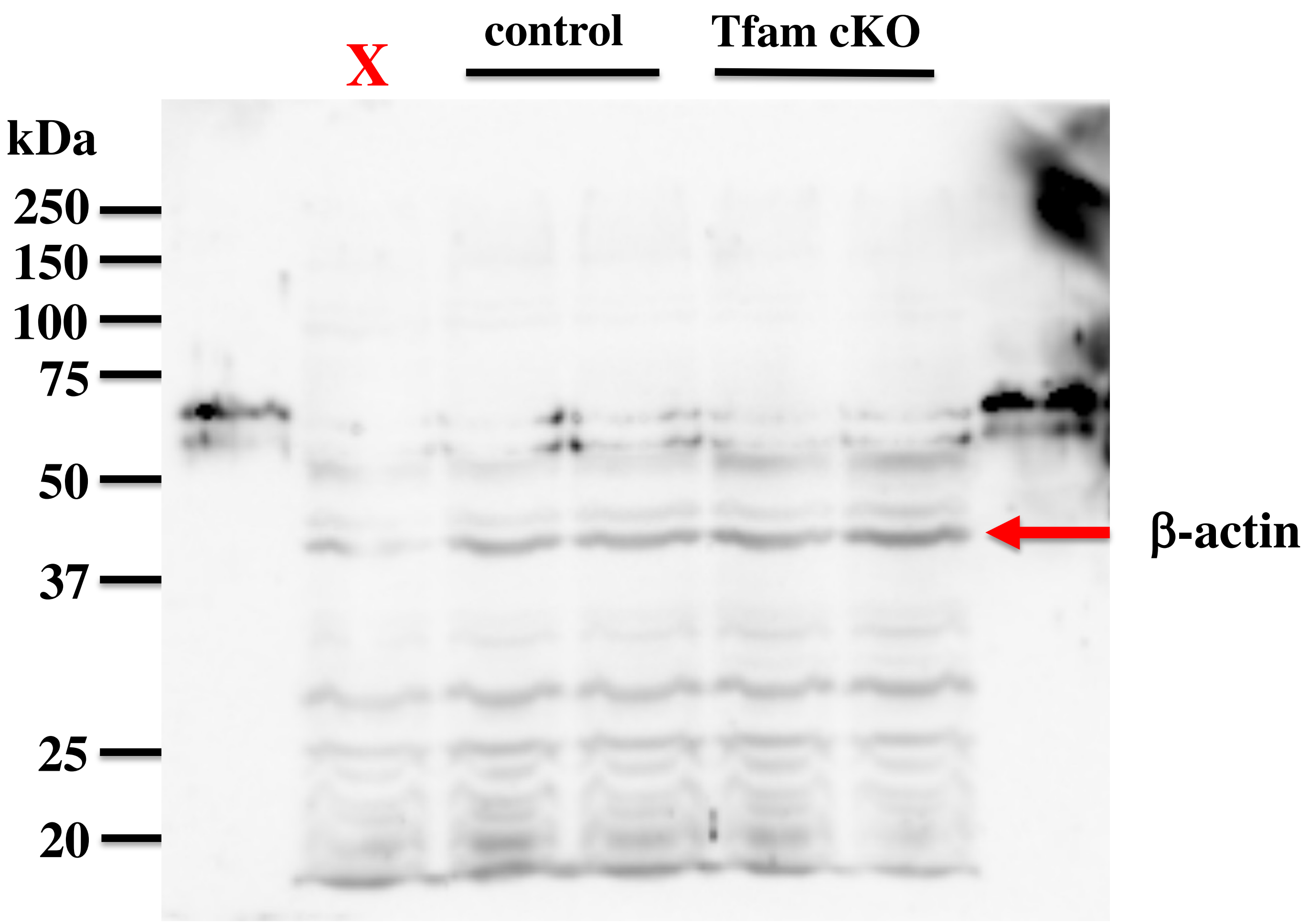

Figure 6A: P0 brain

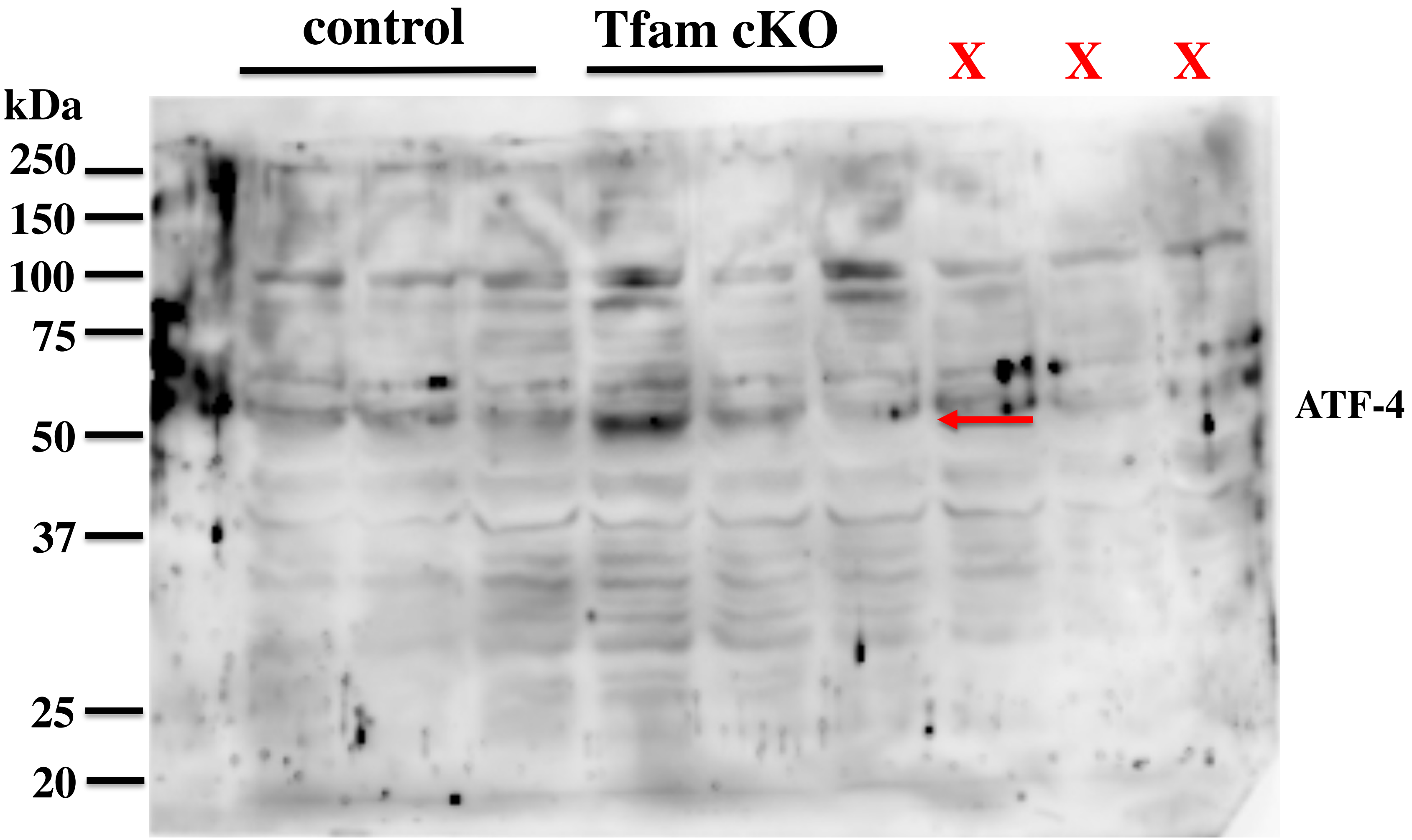

β-actin 1:1000, 4°C O/N

Figure 6A: P0 brain

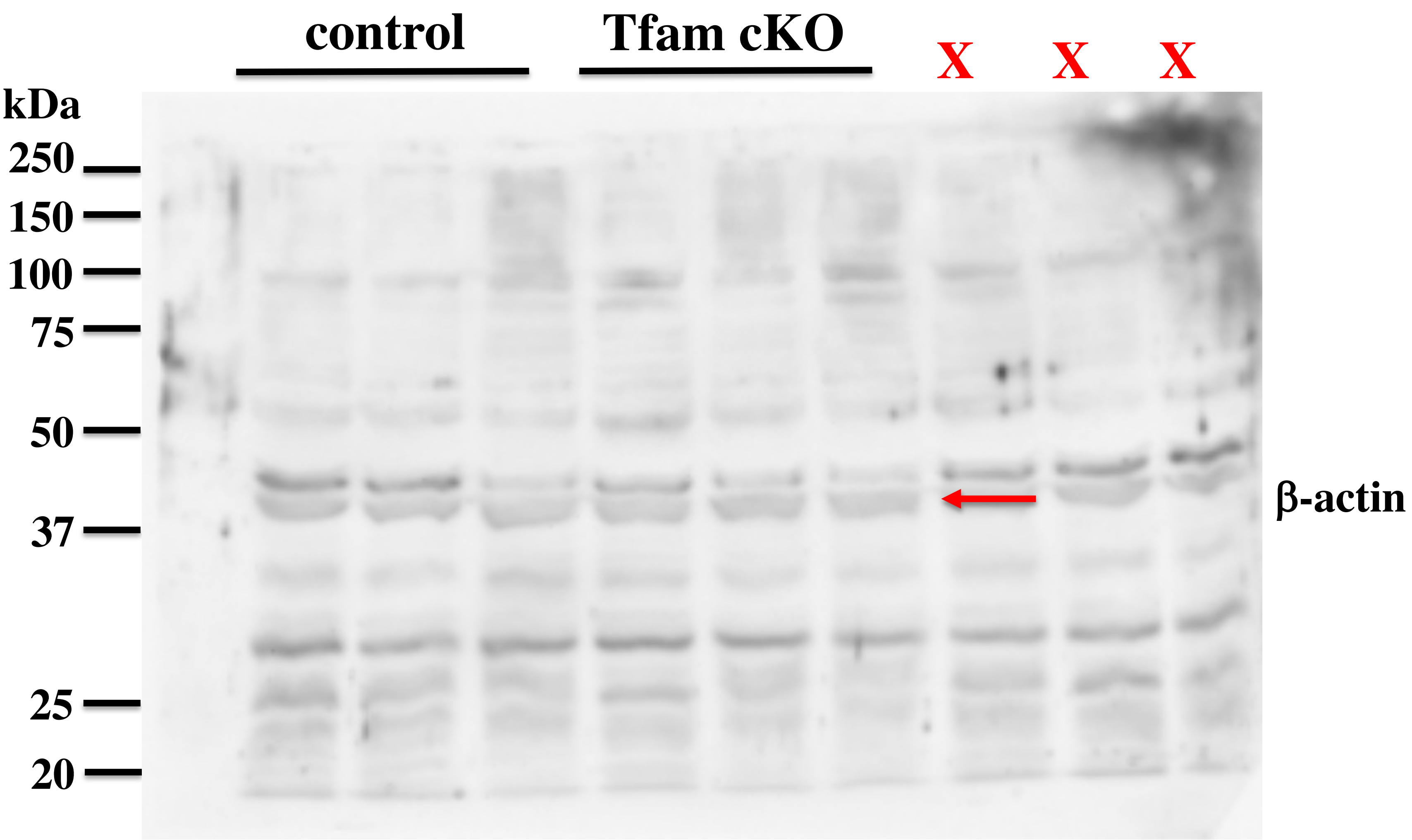

Figure 6: P8 brain

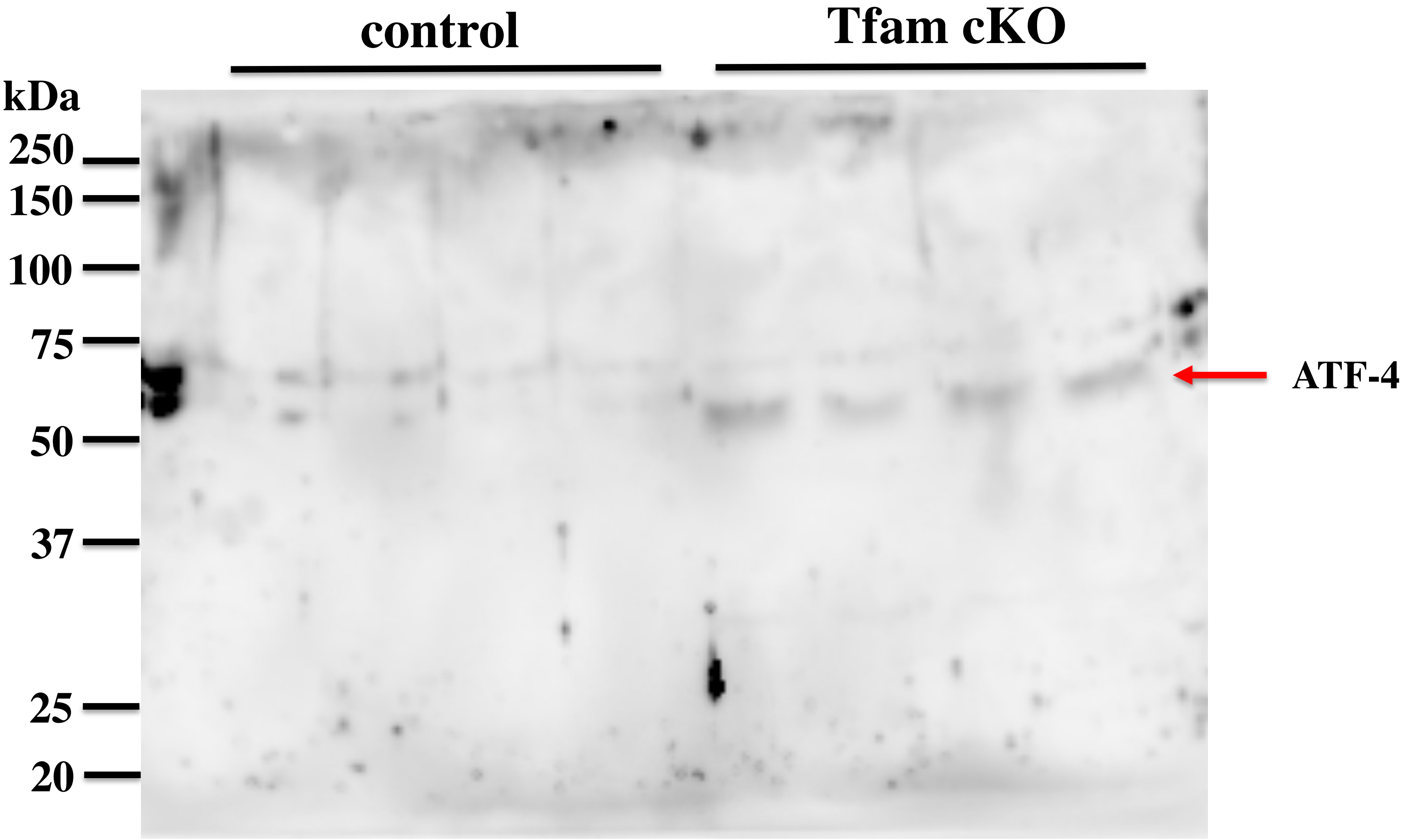

Figure 6A: P8 brain

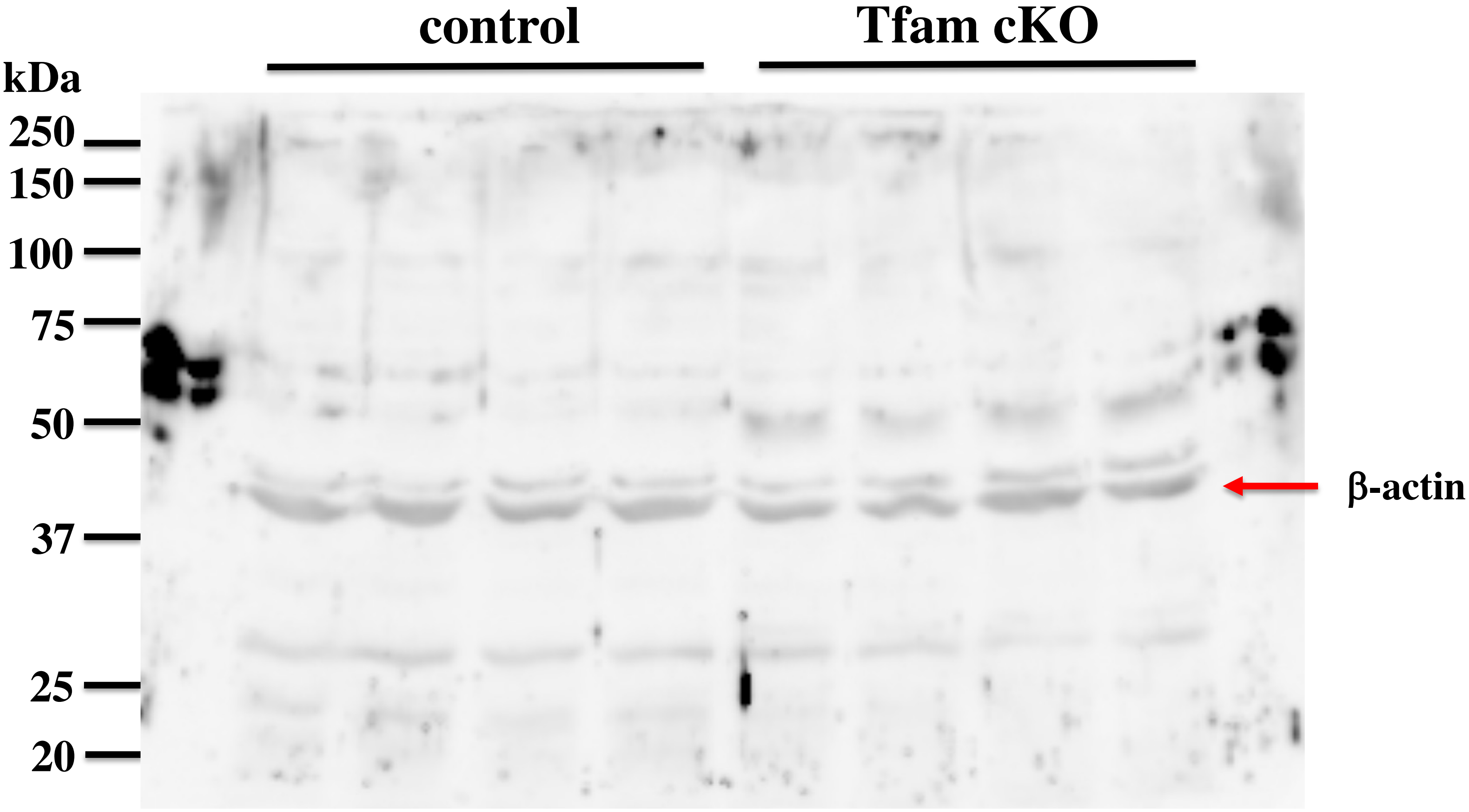

Supplementary Figure 2

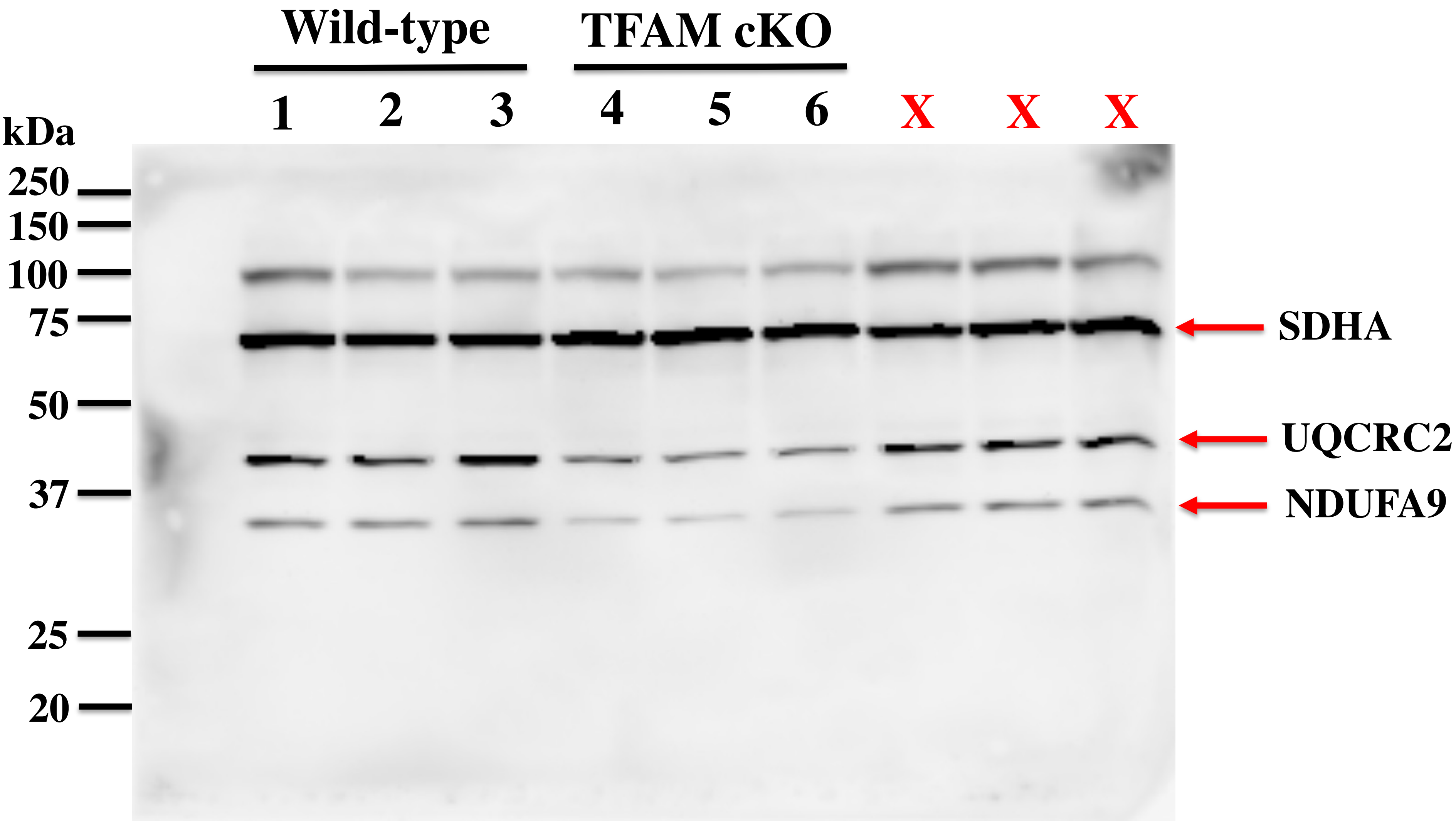

Supplementary Figure 2

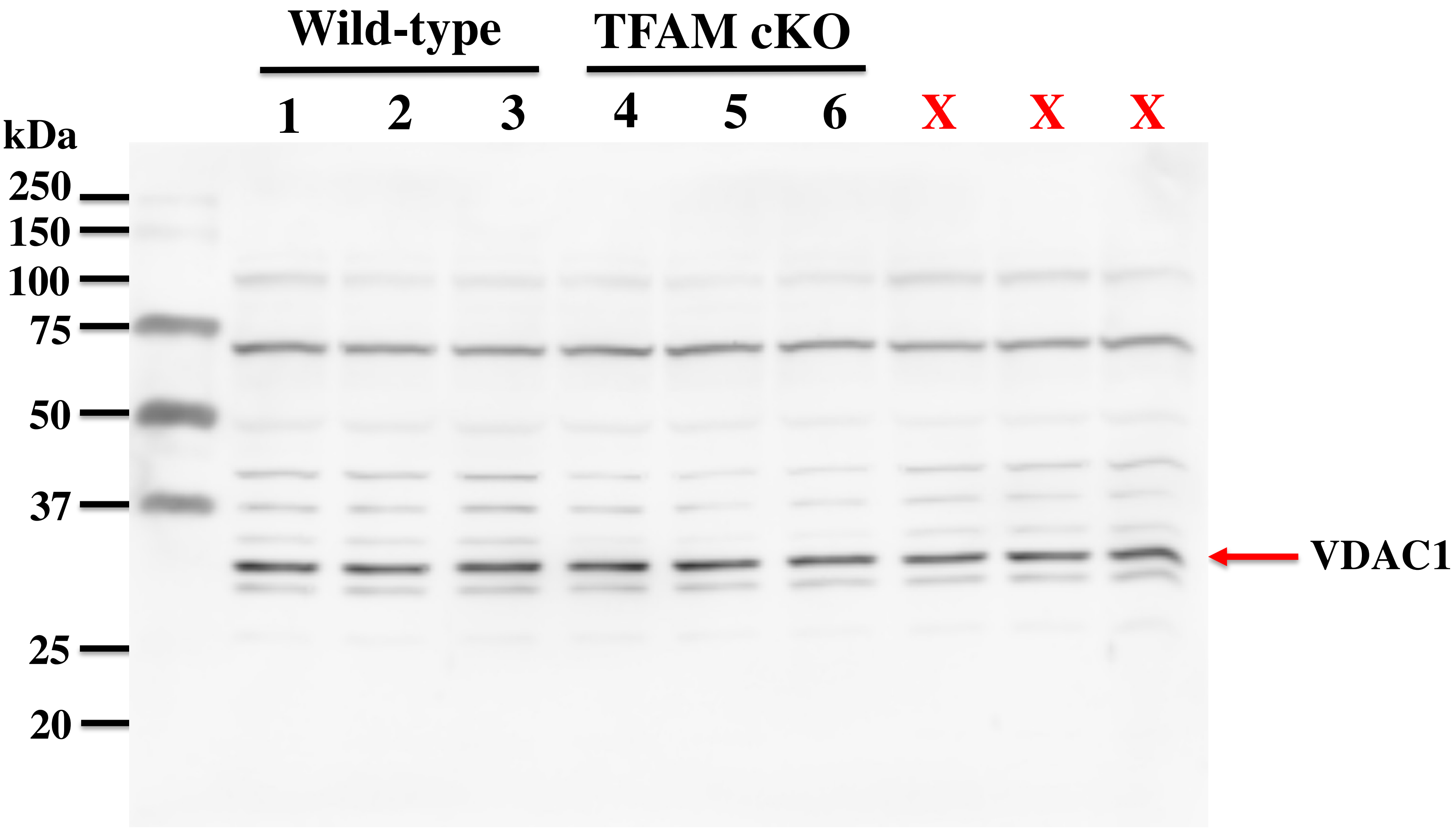

Supplement: S1 Raw images — (PDF) [file pone.0255355.s005.pdf]
